# Mapping the knowledge landscape of planarian regeneration: a century of bibliometric insights

**DOI:** 10.1186/s13062-026-00906-0

**Published:** 2026-07-17

**Authors:** Xinyang Wu, Fang Zhan, Xiaoran Zhang, Haoyang Zhang, Ya Wang, Ting Wang

**Affiliations:** 1 National Demonstration Center for Experimental Basic Medical Education, School of Basic Medicine, Tongji Medical College, Huazhong University of Science and Technology, Wuhan, Hubei 430030 China; 2 Department of Pathogen Biology, School of Basic Medicine, Tongji Medical College, Huazhong University of Science and Technology, Wuhan, Hubei 430030 China; 3 Innovation Research Center, School of Basic Medicine, Tongji Medical College, Huazhong University of Science and Technology, Wuhan, Hubei 430030 China

**Keywords:** Planarian, Stem cell, Regeneration, Bibliometric, Knowledge mapping

## Abstract

**Background:**

Despite over two centuries of research, the knowledge landscape of planarian regeneration—a pivotal model for stem cell biology and regenerative medicine—remains fragmented, hindering interdisciplinary integration and translational progress. To address this gap, we conducted the first large-scale bibliometric analysis integrating machine learning-enhanced burst detection, hierarchical clustering analysis, and cross-disciplinary network mapping.

**Results:**

We systematically analyzed 1,685 publications (1900–2025) from the Web of Science Core Collection, constructing a high-resolution knowledge map that reveals the field’s dynamic evolution. Our analysis identifies three distinct phases: initial morphological exploration (1900–2010), molecular mechanism elucidation (2011–2020), and the current era of interdisciplinary convergence (2021–2025). The United States leads in output and global collaboration, while China ranks third but exhibits limited international engagement. Burst detection highlights emerging frontiers in stem cell regulation, environmental toxicology, and computational modeling. Hierarchical clustering and discipline overlay mapping further uncover convergence between developmental biology and environmental science, neuroscience, education, and engineering. By constructing a high-resolution knowledge map, we identify underexplored thematic intersections suggested by publication trends, thereby providing a data-driven roadmap to navigate future research priorities.

**Conclusion:**

This work provides a structured synthesis of planarian regeneration research, offering a data-driven roadmap to inform future studies, while illustrating how bibliometric approaches can complement traditional literature reviews.

**Supplementary Information:**

The online version contains supplementary material available at https://doi.org/10.1186/s13062-026-00906-0.

## Introduction

Model organisms play an indispensable role in advancing our understanding of the fundamental principles of life. They have substantially contributed to elucidating diverse biological processes, ranging from basic cellular mechanisms to the complexities of health and disease. Notably, numerous Nobel Prizes have recognized groundbreaking discoveries achieved through research on experimental systems, including *Tetrahymena*, *Caenorhabditis elegans*, and *Drosophila melanogaster* etc [1]. In addition, several study systems have been instrumental in clarifying the mechanisms of tissue and organ repair or regeneration. Classical examples such as *Danio rerio* [2, 3], *Notophthalmus viridescens* [4, 5], and *Xenopus laevis* [6, 7] demonstrate the ability to regenerate specific organs or structures through resident stem cells. By contrast, mammalian species such as *Mus musculus* [8, 9] generally require external induction to achieve regeneration, thereby offering critical insights into the induction and regulation of regenerative potential. Nevertheless, the regenerative phenomena observed in these systems remain insufficient to fully explain whole-organ regeneration in vivo. Due to their extraordinary regenerative capacity [10, 11], planarians have attracted increasing attention and have emerged as a unique and indispensable model for investigating regenerative mechanisms and stem cell biology. This prominence is primarily driven by their ability to reconstruct entire organisms from minute tissue fragments. Such biological plasticity is sustained by a highly tractable and abundant population of adult pluripotent stem cells (neoblasts). Furthermore, the availability of highly consistent and scalable regeneration assays offers a robust platform for the precise temporal and spatial dissection of regeneration.

Freshwater planarians, belonging to the class *Turbellaria* within the phylum *Platyhelminthes*, are widely distributed in unpolluted freshwater environments such as streams and ponds. Remarkably, they can regenerate a complete organism from tissue fragments as small as 1/297 of their original body size [10], earning them the description of being “almost immortal under the edge of the knife” [12]. While distinct from the life-cycle reversal observed in organisms like *Turritopsis dohrnii* (the immortal jellyfish), the planarian’s capacity for perpetual tissue renewal and whole-body regeneration provides a uniquely tractable system for studying somatic stem cell dynamics and morphostasis [13]. This extraordinary regenerative capacity is mediated by neoblasts, which account for approximately 20–30% of the total cell population [14, 15]. As the only cell type capable of both proliferation and differentiation, neoblasts play a pivotal role in maintaining tissue homeostasis and coordinating regeneration [15–18]. Moreover, up to 85% of genes implicated in planarian regeneration have homologs in other animals, indicating a high degree of evolutionary conservation. Rather than merely sharing genetic sequences, these homologs govern fundamentally similar biological processes across species. For instance, key regulatory pathways governing axis patterning, cell division, and tissue differentiation exhibit strong functional homology between planarians and mammals (Table S1). Such genetic conservation provides critical insights into stem cell regulation and regenerative mechanisms in mammals [19, 20]. Consequently, planarians have emerged as an exceptional experimental platform for investigating stem cell biology, tissue regeneration, and organ development.

**Fig. 1:**
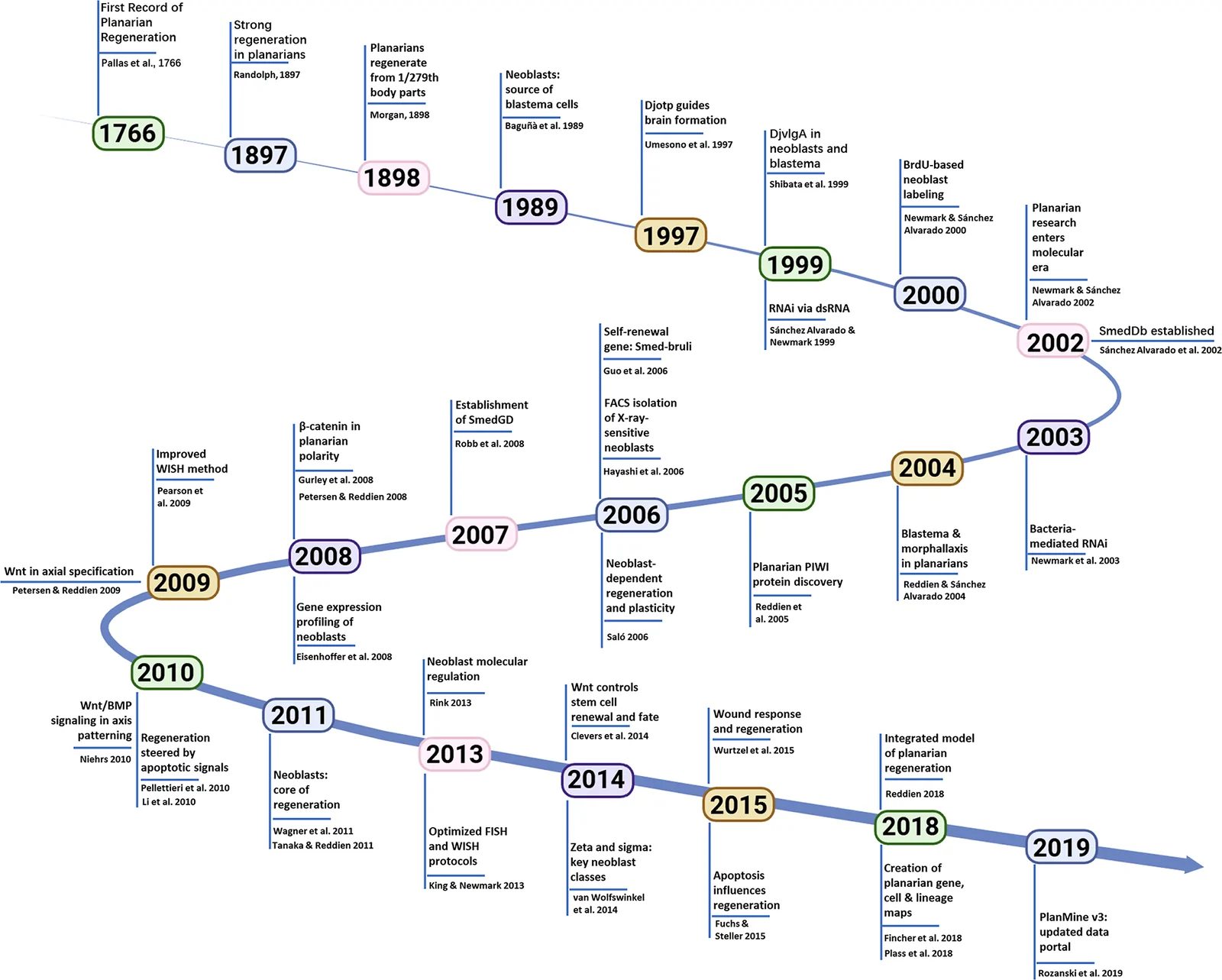
Selected milestones in planarian regeneration research

Research on planarians has spanned more than two centuries, and in recent decades this field has expanded rapidly, yielding numerous significant discoveries (Fig. 1). The earliest description of planarian regeneration was provided by Pallas in 1766 [21]. Subsequently, Randolph [11] and Morgan [10] demonstrated their extraordinary regenerative capacity through amputation–regeneration assays. Building on these foundational observations, research efforts gradually shifted toward elucidating the underlying mechanisms. In 1989, neoblasts were identified as the sole stem cell population and the source of blastema cells [15], thereby establishing the cellular basis of regeneration. Later studies confirmed the totipotency and clonal expansion capacity of neoblasts [16, 17], reinforcing their central role. In 2008, landmark studies by Gurley et al. [22]. and Petersen et al. [23]. revealed that β-catenin is essential for anterior–posterior axis specification during regeneration. This was followed by the discovery of pivotal roles of Wnt and BMP signaling in body axis patterning [24, 25]. Moreover, cell death–associated and injury-induced signaling were shown to significantly influence regenerative outcomes [26–29]. As the field advanced, a more integrated mechanistic view of regeneration began to emerge. A major conceptual step occurred in 2018 when Reddien [30] proposed a model linking positional information with stem cell function, providing a comprehensive framework that incorporates stem cell differentiation, tissue formation, and positional information reconstruction. In parallel, the continuous refinement of experimental techniques [31–34] and the establishment of comprehensive databases [20, 35, 36] have further accelerated progress in dissecting the mechanisms and exploring the broader applications of planarian regeneration.

Although research on planarians has advanced with the development of biotechnology and holds considerable promise, our understanding of this organism—particularly its regenerative capacity—remains limited compared with other well-established systems. This limitation may stem from the lack of a comprehensive knowledge graph in the field, which has resulted in fragmented or even missing connections among researchers from different disciplines. Existing review articles summarize previous findings, but they rarely capture the holistic, dynamic evolution and the interconnected knowledge network of planarian research [37]. To address this gap, we aim to systematically analyze the scientific literature on planarians and construct a structured knowledge graph. Such an approach will facilitate interdisciplinary integration, promote broader engagement, and enable the application of advanced methodologies. This synthesis may help researchers identify under-explored areas and collaboration opportunities, although the ultimate scientific progress will depend on experimental validation and hypothesis-driven research.

Bibliometric is a novel and effective method for analyzing large-scale scientific literature. By employing scientific information visualization techniques, publications will be analyzed both quantitatively and qualitatively to reveal the dynamics, trends, epistemic evolution and the distribution of scientific impact [37–41]. In this study, publications related to planarian and regeneration were retrieved and filtered from the Science Citation Index Expanded (SCIE) database within the Web of Science Core Collection. A comprehensive analysis of key publication information, including country, institution, journal, author, keywords and reference, was conducted to clarify research fronts and epistemic trajectory. Our aim is to construct domain-specific knowledge graphs based on existing publications, in order to enhance understanding of planarian research and provide valuable insights into the dynamics of this domain for scientists form diverse fields. Ultimately, this bibliometric synthesis is intended to inform strategic research planning and interdisciplinary collaboration within the planarian community.

## Methods

### Data source

In this study, literature was retrieved from the Web of Science Core Collection, specifically the Science Citation Index Expanded (SCIE) and the Social Sciences Citation Index (SSCI). These databases encompass high-quality research in both the natural and social sciences, thereby ensuring the comprehensiveness and reliability of the data.

Given the interdisciplinary nature of research on the developmental trajectory of planarian stem cells—where stem cell studies are primarily situated within the natural sciences, while planarians, as classical model organisms in regeneration research, also hold significant importance in life science education—it was essential to select a data source that adequately covers both domains. The Web of Science, widely recognized in the academic community, fulfills this requirement by providing rigorously peer-reviewed and editorially curated literature [42, 43]. Moreover, it is regarded as one of the most suitable databases for bibliometric analysis [44], and its data format is highly compatible with visualization tools such as VOSviewer and CiteSpace [45]. Therefore, both SCIE and SSCI were employed in this study to ensure the comprehensiveness, reliability, and robustness of the bibliometric results.

### Search strategy and keyword cleansing

We performed a topic-based search using the keywords “Planaria,” “Neoblasts,” and “Regeneration.” The specific search strategy was defined as: TS = ((“Planarian” OR “Planarians” OR “Planaria”) AND (“Stem cells” OR “Stem Cell” OR “Mother Cells” OR “Mother Cell” OR “Progenitor Cells” OR “Progenitor Cell” OR “Colony-Forming Unit” OR “Colony Forming Unit” OR “Colony-Forming Units” OR “Colony Forming Units” OR “Neoblasts” OR “Neoblast” OR “Adult Stem Cells” OR “Adult Stem Cell” OR “Somatic Adult Stem Cells” OR “Somatic Stem Cells” OR “Somatic Stem Cell” OR “Adult Somatic Stem Cells” OR “Regeneration” OR “Regenerations” OR “Endogenous Regeneration” OR “Repair” OR “Growth”)). The publication date was restricted to the period from January 1, 1900, to June 17, 2025. Only documents classified as “Article” or “Review” and written in English were included. The search was completed on June 17, 2025, to minimize potential discrepancies caused by subsequent database updates.

**Fig. 2:**
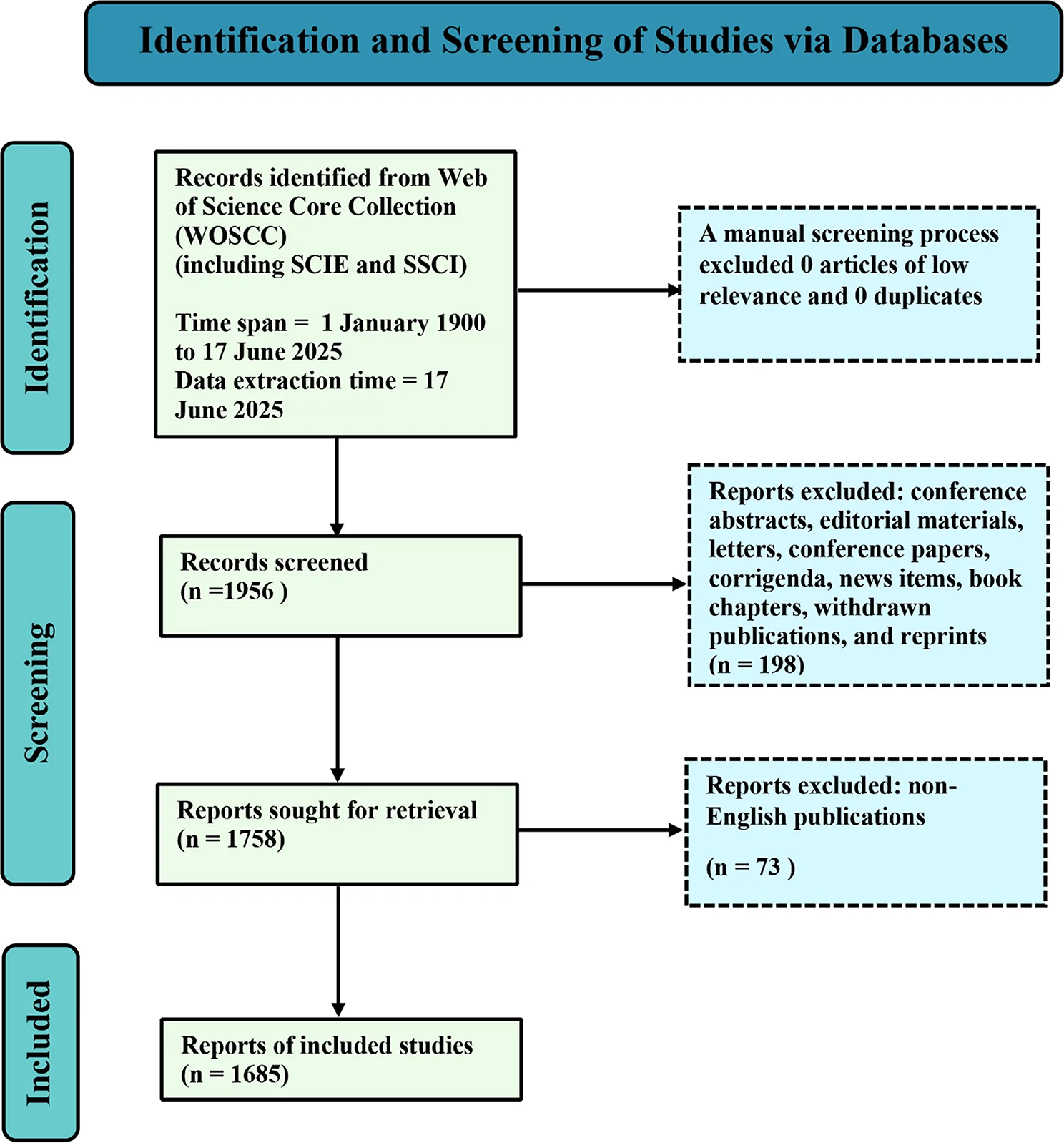
PRISMA flowchart for including studies to review. Limitations on search terms and publication dates yielded 1956 publications. Further restrictions on publication type and language resulted in a final selection of 1685 publications for following bibliometric analysis

Following the PRISMA guidelines [46], we implemented a three-stage screening process, which reduced the initial search results to 1,685 articles eligible for further analysis. Duplicate records were identified and removed using CiteSpace. The overall study framework is presented in Fig. 2. To illustrate our screening process more clearly, we have also included Supplementary Table S2.

To ensure both precision and completeness in keyword selection, we performed a keyword-cleaning procedure that included the consolidation of synonyms, aliases, and singular/plural forms. This process was independently conducted by two authors and subsequently cross-verified. Any discrepancies or unresolved issues were adjudicated by a senior author to ensure consistency. We manually screened strongly relevant keywords and standardized these high-frequency keywords to enhance methodological transparency and reproducibility. The specific keyword merges are provided in Supplementary Table S3. It should be noted that keyword standardization was omitted only in VOSviewer; all other analytical steps underwent standardization. The decision to retain original keywords in VOSviewer was based on two main considerations.

1. VOSviewer’s clustering algorithm relies on co-occurrence frequencies and has built-in tolerance for term variants (e.g., singular/plural forms), depending more on network structures than exact string matching.
2. This approach allowed us to adopt complementary methodological strategies between the two tools: standardization in CiteSpace for burst detection versus preservation of raw term diversity in VOSviewer for network visualization.

This complementary strategy enhances the robustness of our bibliometric analysis by capturing different aspects of the data.

### Technical implementation of bibliometric analysis

Bibliometric data were extracted from the Web of Science Core Collection (WoSCC), including information on authors, titles, journals, publication years, countries/regions, institutions, keywords, citations, abstracts, and references. All data were saved in plain text format. CiteScore values were obtained from the 2024 Journal Citation Reports in the Scopus database. Analytical tools used in this study included Microsoft Office Excel, CiteSpace (v6.3.R3), VOSviewer (v1.6.20), the Bibliometrix package (v4.3.0) in R (v4.4.2), and the ggplot function.

**Fig. 3:**
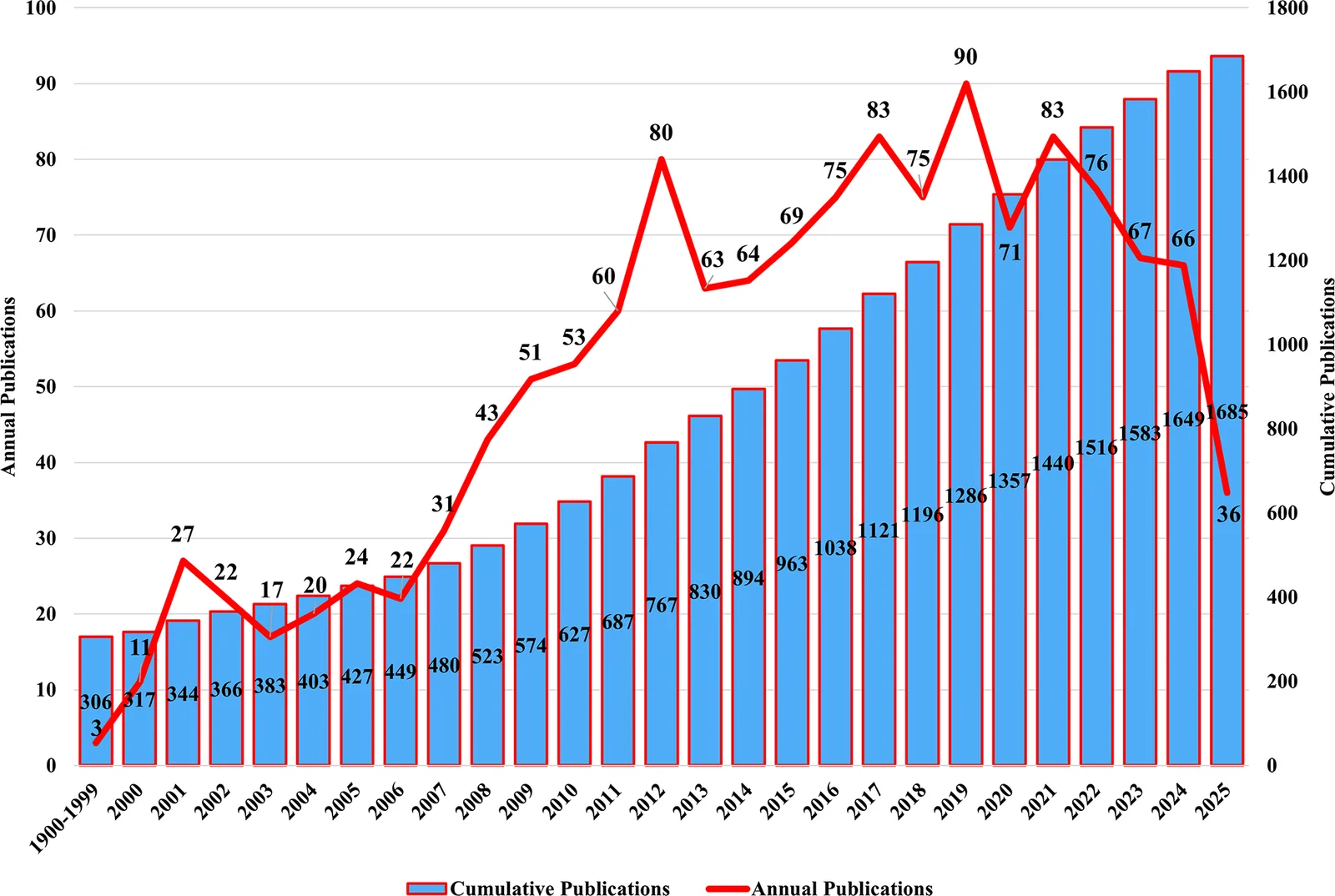
Annual and cumulative output of planarian stem cell and regeneration research publications from 1900 to 2025. The curve shows the overall trend in the number of publications in this field of research and the timing of the surge

**Fig. 4:**
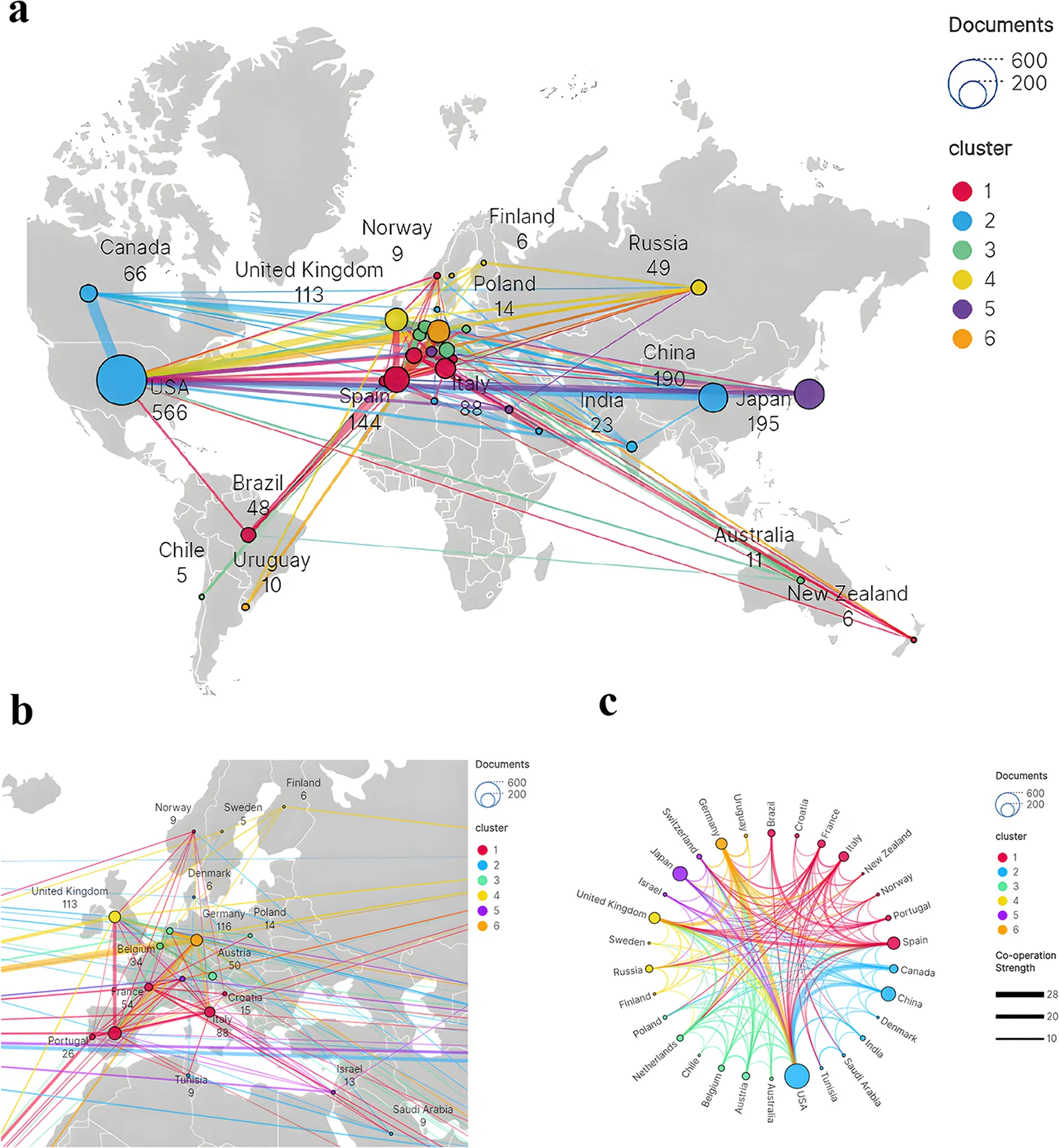

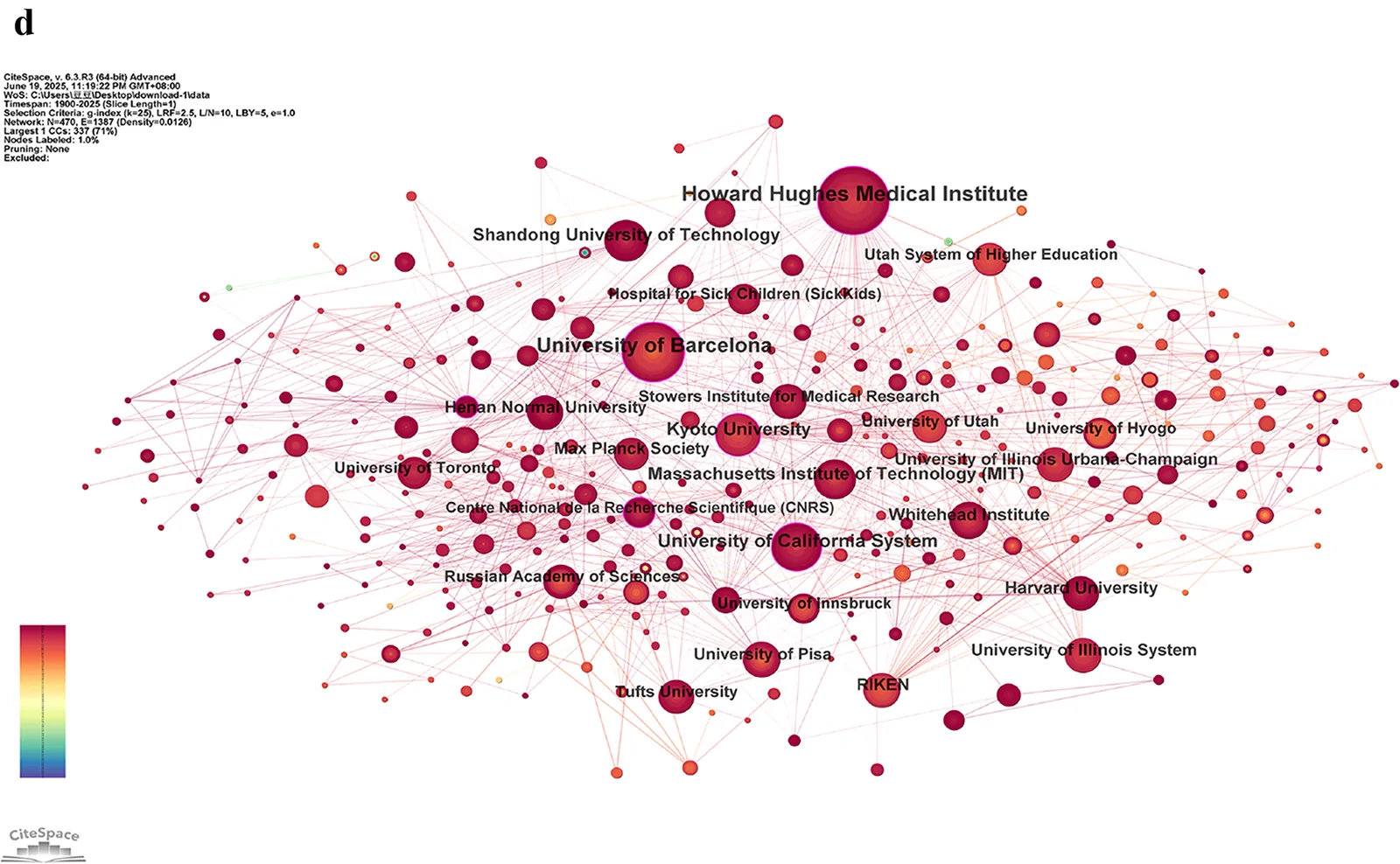
Analysis of country and international cooperation. Figure **a-c** is country co-authorship analysis. The size of each circle indicates the number of documents produced by that country. The different colors of the circles represent different clusters, and the lines connecting them indicate cooperative relationships, with the thickness of them indicating the strength of the cooperation. (**a**) Country co-authorship overlay visualization map. (**b**) This image is a close-up of Fig. 4a locality. (**c**) Country co-authorship analysis diagram. (**d**) Institutional collaboration network visualization. 24 institutions are marked. The size of the circle represents the extent of the institution’s contribution, and the halo around the circle indicates the institution’s close cooperation with other institutions

To improve the accuracy and representativeness of the analysis, data preprocessing was carried out according to the requirements of each tool. This process included merging variant names of the same institution, grouping sub-institutions under parent institutions, and standardizing keywords with similar or identical meanings. The following summarizes the software applied and the major analytical steps undertaken to ensure methodological rigor:

1. A milestone map (Fig. 1) was created using BioRender and PowerPoint 2021.
2. Annual publication volume and proportion (Fig. 3) were plotted with Microsoft Office Excel 2021.
3. Co-authorship networks and keyword co-occurrence maps were constructed and visualized using VOSviewer (v1.6.20). In these maps, node size reflects publication volume, line thickness indicates collaboration strength, and color denotes clusters. For country-level collaboration (Fig. 4a, b and c), data from VOSviewer were converted into GML format and visualized with SCImago Graphica. A minimum threshold of five publications was applied, resulting in the inclusion of 30 countries/regions. Keyword clustering and subject distribution (Fig. 6) were visualized using a combination of VOSviewer and Pajek.
4. Institutional collaboration and burst detection analyses were conducted using CiteSpace (v6.3.R3), a widely adopted tool in bibliometric research [47]. Burst detection included all keywords with a frequency ≥ 1 between 2000 and 2025, with a burst strength threshold > 2 to identify emerging research hotspots. Parameters for institutional collaboration analysis were as follows: time span (1900–2025), time slice (1 year), g-index (k = 25), LRF = 2.5, LBY = 5, e = 1.0.
5. Thematic evolution analysis was performed using the Bibliometrix package (v4.3.0) in R (v4.4.2). The timeline was divided into three intervals (2005, 2014, and 2019) to trace thematic dynamics and evolving research trends. A heatmap of core keywords was generated with the ggplot function based on CSV-formatted data.

### Bibliometric analysis methods

This study applied a range of bibliometric methods to identify trends and emerging directions in planarian stem cell research. The specific approaches were as follows:

Citation Analysis: Citation frequency was used as the primary metric to evaluate the academic influence of articles, journals, and authors. On this basis, high-impact publications and core references were identified. For the top 20 authors, we additionally calculated a local H‑index restricted to citations within our dataset, following the standard H‑index definition (H ≤ N) [48].

Collaboration Analysis (Authors, Countries, Institutions): Collaboration networks among researchers were examined using CiteSpace and VOSviewer, which enabled visualization of collaboration patterns across authors, institutions, and countries [49].

Keyword Co-occurrence Analysis: Thematic structures and interdisciplinary linkages were explored by analyzing keyword co-occurrence [50]. VOSviewer was primarily employed for clustering analysis to identify key topics and research trends.

Burst Detection: Burst detection was applied to identify topics that exhibited sudden increases or decreases in scholarly attention. A “burst” refers to a sharp change in activity or frequency of an element over a defined period. This method helps to highlight emerging concepts, potential future directions, and previously active or pioneering nodes within the field [37].

Journal Metrics Analysis: Journal impact was assessed on the basis of publication volume, citation counts, and impact indicators [51].

## Research ethics

This study was conducted as a bibliometric analysis, with all data obtained from publicly accessible online sources. As no human participants or animals were involved, approval from an institutional review board was not required.

### Use of AI-assisted technology

During the preparation of this work, the authors utilized ChatGPT (OpenAI, GPT-4.1 mini) for language editing purposes, specifically to check grammar, optimize word choice, and improve sentence flow. Following the use of this tool, the authors thoroughly reviewed and edited the output as needed. The authors are solely responsible for the content of the published work, having critically evaluated and endorsed all textual material.

### Phase delineation and growth rate analysis

For phase delineation, we calculated the annual growth rate (AGR) of publication volume: AGR(y) = [N(y) – N(y − 1)) / N(y − 1)] × 100%, where N(y) is the number of publications in year y. A 5-year moving average rate was applied to smooth short-term fluctuations. Inflection points were defined where the moving average AGR showed a sustained change in trend direction for at least three consecutive years. Annual growth rates and 5-year moving averages of publications are compiled in Supplementary Table S4.

## Results

### Time trend of the publications

To illustrate publication trends in the field of planarian stem cells and regeneration, annual publication counts were analyzed with a line chart, and cumulative publications were represented with a bar chart in Excel (Fig. 3). Around 2010, the 5-year moving average growth rate of publication output reached approximately 20%; prior to that, this rate exhibited considerable volatility. The year 2020 likewise marked another critical juncture, after which the 5-year moving average growth rate trended toward negative values (Fig. S1). Based on the growth rate analysis described in the Methods section, the quantitative criterion identified 2010 and 2020 as the two major inflection points, thereby segmenting the publication history from 1900 to 2025 into three distinct phases:

#### Phase I (1900–2010): Initial exploration

Although research began in the early 20th century, progress remained slow for decades. After 2000, publication activity gradually increased, with annual output stabilizing at 30–50 articles between 2007 and 2010. This phase was characterized by weak research foundations, limited by underdeveloped experimental techniques, model systems, and molecular tools. As a result, stem cell mechanisms underlying planarian regeneration had not yet become a mainstream research focus, and most studies relied on macroscopic observations, descriptive biology, and empirical validation.

#### Phase II (2011–2020): Rapid expansion

From 2011 onward, advances in molecular biology and genomics, along with the growing recognition of planarians as a model system for regeneration, drove a substantial increase in publications. Annual output reached 80 in 2012 and peaked at 90 in 2019, with a slight decline in 2020 attributable to the COVID-19 pandemic. During this phase, yearly publication counts consistently exceeded 60. Research emphasis shifted toward pluripotency, molecular regulatory mechanisms, and gene expression profiling of neoblast stem cells, reflecting a transition to cellular, molecular, and bioinformatics-driven approaches with increasing interdisciplinary integration [17, 52].

#### Phase III (2021–2025): Post-pandemic deceleration

Although publication numbers remained high in 2021 (83 articles), a gradual decline followed, based on the data collected up to June 17, the annual publication volume is on track to reach approximately 60 articles by the end of the year 2025. While the pandemic stimulated integration with applied areas such as environmental toxicology and drug screening, it also introduced disruptions through laboratory closures and delayed timelines. As a result, growth slowed or plateaued. Nevertheless, the cumulative number of publications reached 1,685 by 17 June 2025, underscoring the established role of planarian research in stem cell biology, regenerative medicine, and model organism studies [53].

Overall, this field has experienced a trajectory of slow emergence, rapid growth, and mild deceleration. Research content has evolved from superficial description to mechanistic depth, and from a single-model focus to multi-system exploration. With sustained interdisciplinary engagement spanning environmental science, bioinformatics, and materials engineering, planarian research continues to hold considerable potential for multidisciplinary development.

### Analysis of countries/regions and organizations cooperation

As shown in Fig. 4a, b and c, the United States leads the field in terms of publication output, scope of collaboration, and strength of partnerships. With 190 publications, China ranks third after the United States and Japan. However, apart from relatively strong collaboration with the United States (approximately 20%), China’s cooperation with other countries or regions remains limited and generally weaker. Many of the more active countries in this field are located in Europe, with Germany, the United Kingdom, and Italy contributing the largest number of publications. Collaboration among European countries, as well as between Europe and the United States, is relatively frequent and extensive. In North America, both the United States and Canada have made substantial contributions to global research. Within Asia, China and Japan are the leading contributors, with India also playing a role. In South America, Brazil is the primary contributor, while Chile and Uruguay also show some activity. A smaller number of publications originate from Australia and New Zealand in Oceania, and from Tunisia in Africa.

Figure 4d presents a collaboration network generated by CiteSpace, illustrating the relationships among global research institutions. Prominent nodes such as *Harvard University*, *University of Barcelona*, and the *University of California System* highlight their central roles in international collaboration. Chinese institutions, including *Shandong University of Technology* and *Henan Normal University*, are also visible, reflecting China’s increasing importance in global research partnerships. The color transitions in the network over time further indicate the dynamic evolution and shifting trends of institutional research activity

### Analysis of author

**Table 1:** Top 20 authors with the most citations

Rank^a)^	Author	Citations^b)^	Documents	Average citation per paper	H-Index^c)^	Affiliation^d)^
1	Alvarado, A. Sanchez	8383	67	125.12	36	Stowers Institute for Medical Research
2	Reddien, Peter W.	5744	54	106.37	34	Whitehead Institute
3	Agata, Kiyokazu	5657	98	57.72	40	Graduate University for Advanced Studies - Japan
4	P. A. Newmark	5059	52	97.29	28	University of Wisconsin Madison
5	Emili Salo	2775	50	55.50	12	University of Barcelona
6	Francesc Cebria	2582	35	73.77	20	University of Barcelona
7	Umesono, Yoshihiko	2078	29	71.66	20	University of Hyogo
8	Jochen C. Rink	1807	19	95.11	13	Max Planck Inst Mol Cell Biol & Genet
9	Michael Levin	1798	39	46.10	13	Tufts University
10	B. J. Pearson	1722	37	46.54	20	University of Toronto
11	Watanabe, Kenji	1693	28	60.46	22	University of Hyogo
12	Baguna, Jaume	1414	22	64.27	17	University of Barcelona
13	Christian P. Petersen	1411	17	83.00	12	Northwestern University
14	Shibata, Norito	1339	21	63.76	15	Tsuyama Coll
15	Wagner, Daniel E	1107	6	184.50	6	Appalachian State University
16	Takeshi Inoue	1083	24	45.13	14	Gakushuin University
17	Orii, Hidefumi	1067	17	62.76	14	University of Hyogo
18	Teresa Adell Creixell	1049	26	40.35	14	Universitat de Barcelona
19	Bartscherer, Kerstin	992	14	70.86	12	University Osnabruck
20	Charles Serhan	918	11	83.45	4	MassGeneral Brigham MGB

^(a)^ Ranked by citation number. Authors with the same number of citations were then ranked by total citations and H-index; ^(b)^ Citations refer to the sum of citations received these years of each author; ^(c)^ In this context, H-index denotes the local H-index. Local H‑index calculated based solely on citations from within the 1,685‑publication dataset. This value satisfies H ≤ N; ^(d)^ Affiliation for each author is the latest one shown on Web of Science in order to distinguish different people only

Received citations and scientific productivity form the basis of many evaluation metrics. To assess author contributions, we ranked the core authors in this field by citation frequency and summarized their information, including names, total citations, number of publications, average citations per paper, and local H-index (Table 1). Professor A. Sánchez Alvarado, with 8,383 citations, is the most highly cited scholar worldwide in this field—nearly 3,000 more citations than the second-ranked author. He has published 67 papers, placing him second globally in terms of output, underscoring his significant contributions. Professor Kiyokazu Agata, with 98 publications, is the most prolific author in the field. Professor Daniel E. Wagner, ranked 15th overall, has the highest average citation rate of 184.5 per paper. Despite having published only six papers—the fewest among the top 20 authors—his work has accumulated 1,107 citations, demonstrating that his contributions have been widely recognized internationally.

### Analysis of journal and article

In the bibliometric analysis of planarian regeneration biology, we examined journal and article citations to trace the academic development of the field and the evolution of research hotspots.

Table 2 lists the top 20 most cited journals. In terms of total citations, *Development*, *Developmental Biology*, and *Science* stand out as the most influential journals. Notably, *Science* has an average of 281 citations per paper, underscoring its impact in the academic community. Meanwhile, journals such as *Cell*, *Cell Stem Cell*, *Developmental Cell*, and *Nature* dominate regenerative biology and stem cell research, reflecting the interdisciplinary integration and significant cross-cutting value of this field.

It should be noted that citation metrics are influenced by multiple factors beyond scientific merit, including journal visibility, publication year, and review article status. Thus, while these metrics provide useful macroscopic insights, they should not be over‑interpreted as direct measures of research quality [54].

**Table 2:** Top 20 journals with the most citations

Rank^a)^	Journal	Citations	Documents	Citations per document	Citescore 2024^b)^	Publisher
1	DEVELOPMENT	4471	63	70.97	5.7	Company of Biologists Ltd
2	DEVELOPMENTAL BIOLOGY	4170	69	60.43	5.3	Elsevier
3	SCIENCE	3928	14	280.57	48.4	American Association for the Advancement of Science
4	PROCEEDINGS OF THE NATIONAL ACADEMY OF SCIENCES OF THE UNITED STATES OF AMERICA	2732	34	80.35	16.5	National Academy of Sciences
5	DEVELOPMENTAL CELL	2062	15	137.47	16.7	Elsevier
6	DEVELOPMENT GROWTH & DIFFERENTIATION	1915	40	47.88	3.3	John Wiley & Sons
7	DEVELOPMENT GENES AND EVOLUTION	1716	31	55.35	2.5	Springer
8	ELIFE	1651	32	51.59	12.9 (2023)	eLife Sciences Publications Ltd.
9	DEVELOPMENTAL DYNAMICS	1184	21	56.38	5.3	John Wiley & Sons
10	INTERNATIONAL JOURNAL OF DEVELOPMENTAL BIOLOGY	1112	45	24.71	2.1	UPV/EHU Press
11	NATURE	1064	8	133.00	78.1	Springer Nature
12	CELL	984	5	196.80	74.8	Elsevier
13	JOURNAL OF EXPERIMENTAL ZOOLOGY PART A-ECOLOGICAL AND INTEGRATIVE PHYSIOLOGY	942	35	26.91	4.4	John Wiley & Sons
14	CELL STEM CELL	761	5	152.20	32.9	Elsevier
15	ZOOLOGICAL SCIENCE	745	22	33.86	1.8	Zoological Society of Japan
16	PLOS ONE	743	27	27.52	5.4	Public Library of Science
17	COLD SPRING HARBOR PERSPECTIVES IN BIOLOGY	741	9	82.33	14.1	Cold Spring Harbor Laboratory Press
18	PLOS GENETICS	734	21	34.95	7.9	Public Library of Science
19	CELL REPORTS	678	17	39.88	12.9	Elsevier
20	SEMINARS IN CELL & DEVELOPMENTAL BIOLOGY	578	20	28.90	15.2	Elsevier

^(a)^ Ranked by citation number; ^(b)^ All journal metrics presented in this table are CiteScore 2024 values, extracted from the Scopus database (Elsevier) on 17 June 2025. The data represent the citation impact of source publications over a four‑year window (2021–2024), as defined by Elsevier

Table 3 presents the top 20 most cited articles between 2000 and 2024, which represent foundational literature with long-standing influence. For example, *An integral program for tissue renewal and regeneration: Wnt signaling and stem cell control* (Clevers, 2006) and *Fundamentals of planarian regeneration* (Reddien, 2008) deeply explored the link between Wnt signaling and stem cell function, elucidating the multifaceted roles of stem cells in regeneration and providing theoretical support for subsequent studies [55, 56]. Their high citation counts and stable annual citation rates indicate sustained academic interest in gene regulation and stem cell biology, and have fostered cross-disciplinary studies in regenerative and stem cell biology. With continued research, the focus has shifted toward stem cell self-renewal and differentiation mechanisms. For instance, *SMEDWI-2 is a PIWI-like protein that regulates planarian stem cells* (Reddien, 2005) advanced stem cell research by revealing the role of PIWI-like proteins in planarian stem cell regulation [57]. Its growing citation frequency reflects the increasing recognition of stem cells as central players in planarian regeneration.

**Table 3:** Top 20 articles with the most citations

Rank^a)^	Title	First author^b)^	Journal	Publication type	Total Citations	TC per Year^c)^
1	An integral program for tissue renewal and regeneration: Wnt signaling and stem cell control	Clevers, Hans	SCIENCE	Review	1050	87.50
2	Fundamentals of planarian regeneration	Reddien, PW	ANNUAL REVIEW OF CELL AND DEVELOPMENTAL BIOLOGY	Review	564	25.64
3	Live to die another way: modes of programmed cell death and the signals emanating from dying cells	Fuchs, Yaron	NATURE REVIEWS MOLECULAR CELL BIOLOGY	Review	499	45.36
4	Clonogenic neoblasts are pluripotent adult stem cells that underlie planarian regeneration	Wagner, Daniel E	SCIENCE	Article	490	32.67
5	SMEDWI-2 is a PIWI-like protein that regulates planarian stem cells	Reddien, PW	SCIENCE	Article	487	23.19
6	Double-stranded RNA specifically disrupts gene expression during planarian regeneration	Alvarado, AS	PROCEEDINGS OF THE NATIONAL ACADEMY OF SCIENCES OF THE UNITED STATES OF AMERICA	Article	438	16.22
7	The cellular basis for animal regeneration	Tanaka, Elly M.	DEVELOPMENTAL CELL	Review	414	27.60
8	Not your father’s planarian: A classic model enters the era of functional genomics	Newmark, PA	NATURE REVIEWS GENETICS	Review	409	17.04
9	Bromodeoxyuridine specifically labels the regenerative stem cells of planarians	Newmark, PA	DEVELOPMENTAL BIOLOGY	Article	407	15.65
10	Wnt Signaling and the Polarity of the Primary Body Axis	Petersen, Christian P.	CELL	Review	406	23.88
11	Macrophage proresolving mediator maresin 1 stimulates tissue regeneration and controls pain	Serhan, Charles N.	FASEB JOURNAL	Article	390	27.86
12	Comparative Aspects of Animal Regeneration	Brockes, Jeremy R.	ANNUAL REVIEW OF CELL AND DEVELOPMENTAL BIOLOGY	Review; Book Chapter	386	21.44
13	Evolution of animal regeneration: re-emergence of a field	Bely, Alexandra E.	TRENDS IN ECOLOGY & EVOLUTION	Review	386	24.13
14	β-catenin defines head versus tail identity during planarian regeneration and Homeostasis	Gurley, Kyle A.	SCIENCE	Article	376	20.89
15	Apoptotic Cells Activate the “Phoenix Rising” Pathway to Promote Wound Healing and Tissue Regeneration	Li, Fang	SCIENCE SIGNALING	Article	373	23.31
16	Bridging the regeneration gap: genetic insights from diverse animal models	Alvarado, AS	NATURE REVIEWS GENETICS	Review	362	18.10
17	Identification of genes needed for regeneration, stem cell function, and tissue homeostasis by systematic gene perturbation in planaria	Reddien, PW	DEVELOPMENTAL CELL	Article	353	16.81
18	Molecular analysis of stem cells and their descendants during cell turnover and regeneration in the planarian Schmidtea mediterranea	Eisenhoffer, George T.	CELL STEM CELL	Article	323	17.94
19	Advances in understanding tissue regenerative capacity and mechanisms in animals	Poss, Kenneth D.	NATURE REVIEWS GENETICS	Review	313	19.56
20	Cell type atlas and lineage tree of a whole complex animal by single-cell transcriptomics	Plass, Mireya	SCIENCE	Article	308	38.50

^(a)^ Ranked by total citations; ^(b)^ The first authors showed up on Web of Science. This doesn’t mean this author contributed the most; ^(c)^ TC per year means is calculated by dividing the total number of citations by the number of years the author or journal has been publishing papers

Table 4 highlights the top 20 articles with the strongest citation bursts. Early bursts centered on gene regulation and stem cell function. For example, *Identification of Genes Needed for Regeneration*,* Stem Cell Function*,* and Tissue Homeostasis by Systematic Gene Perturbation in Planaria* (Reddien, 2005) identified key genes involved in regeneration, representing a breakthrough that accelerated the application of gene perturbation techniques in planarian research [19]. Other key works, such as *SMEDWI-2 is a PIWI-like protein that regulates planarian stem cells* (Reddien, 2005) and *Molecular Analysis of Stem Cells and Their Descendants during Cell Turnover and Regeneration in the Planarian Schmidtea mediterranea* (Eisenhoffer, 2008), further advanced understanding of stem cell self-renewal and differentiation [57, 58]. The β-catenin pathway also emerged as a pivotal focus, with studies such as *β-Catenin Defines Head Versus Tail Identity During Planarian Regeneration and Homeostasis* (Gurley, 2008) and *Smed-β-catenin-1 Is Required for Anteroposterior Blastema Polarity in Planarian Regeneration* (Petersen, 2008) clarifying its role in axis specification [22, 23]. More recently, approaches such as single-cell analysis have refined understanding of stem cell heterogeneity. For example, *Functionally Distinct Classes within the Planarian Stem Cell Compartment* (van Wolfswinkel, 2014) identified functional subpopulations of stem cells, advancing cell typology research [32].

**Table 4:**
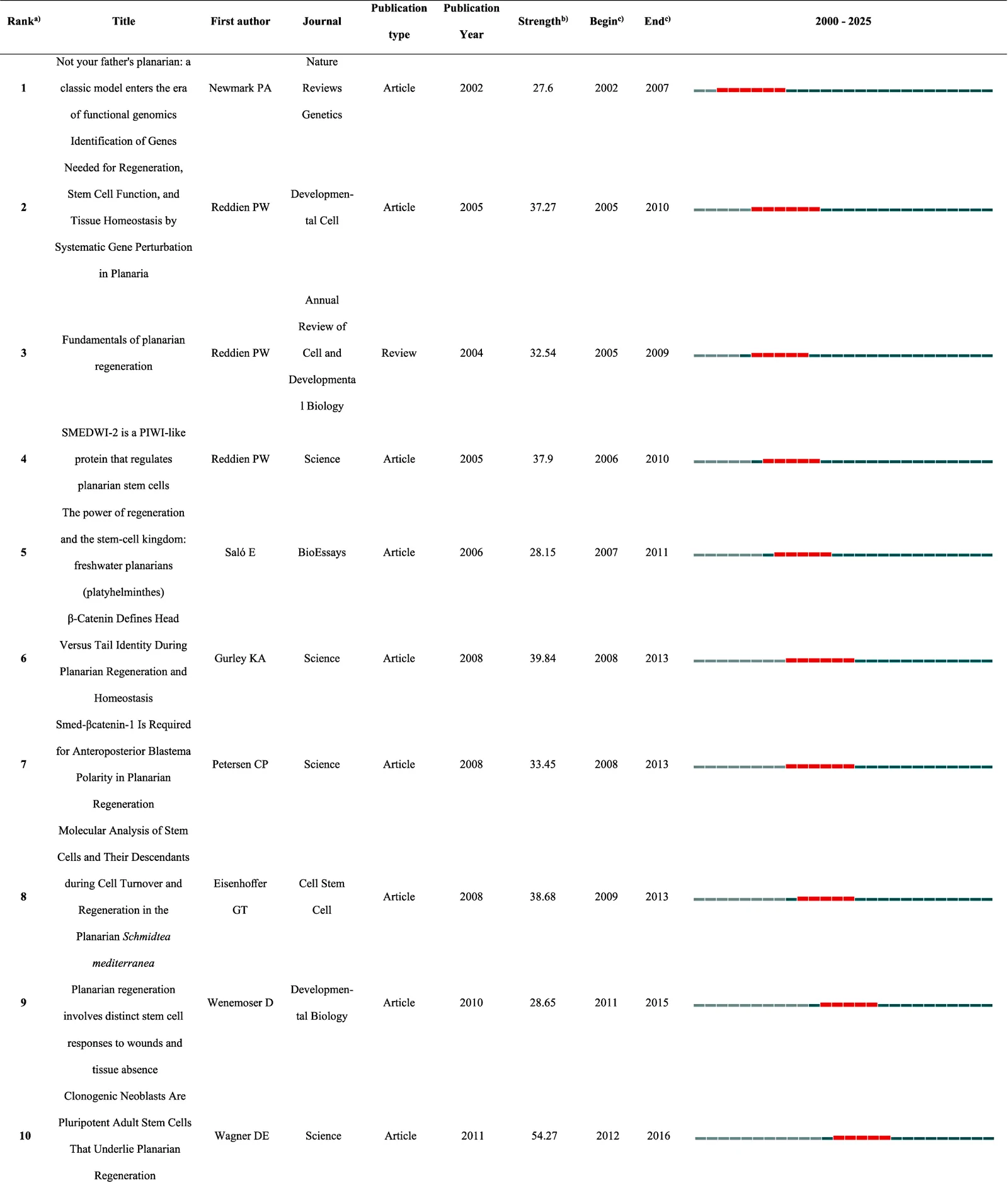

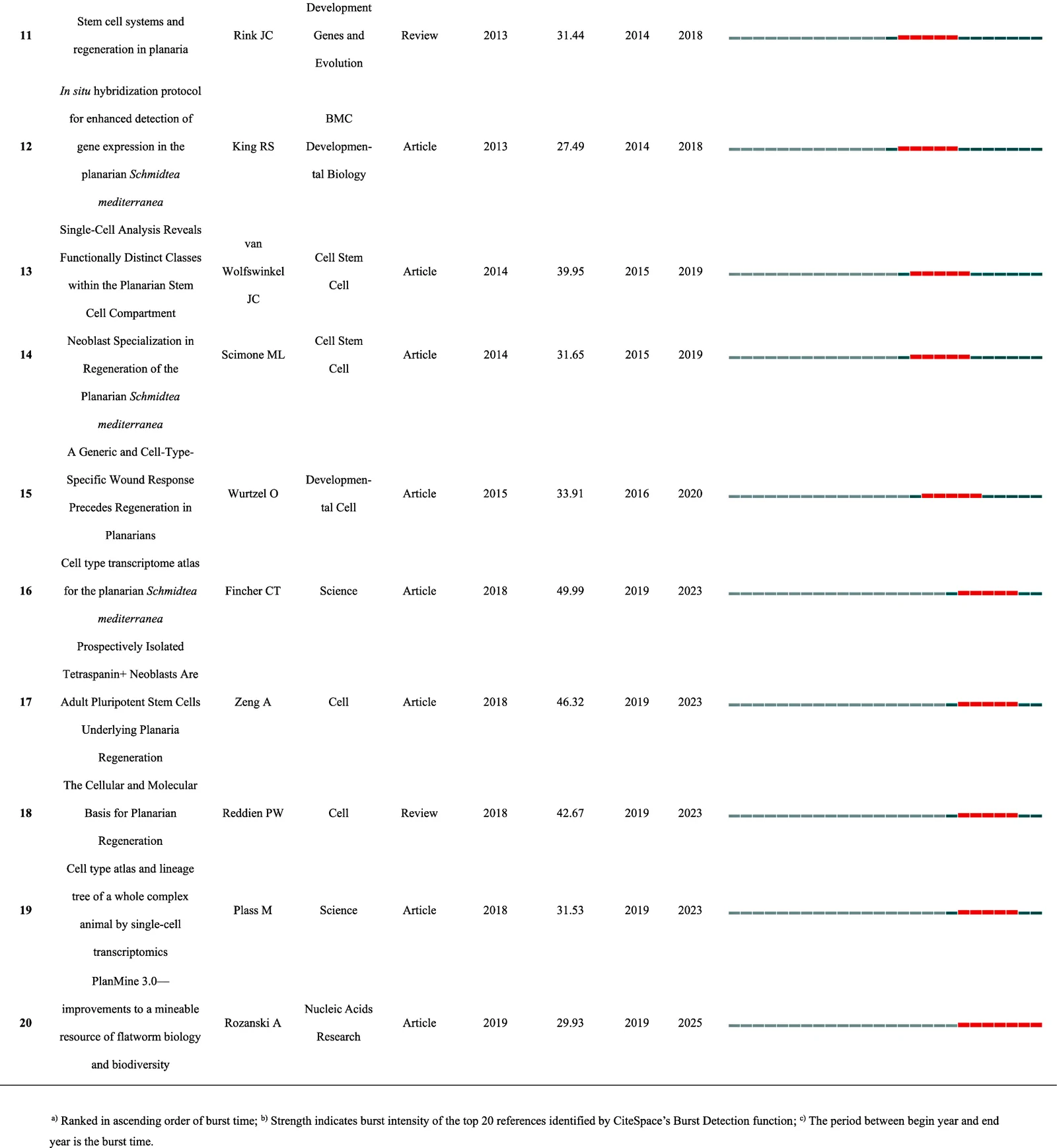
Top 20 References with the Strongest Citation Bursts

Overall, citation analysis shows that planarian regeneration research has gradually shifted from descriptive studies of cell behavior and regeneration biology toward more detailed molecular mechanisms, with particular emphasis on stem cell regulation and gene function. Since 2005, planarians have also been widely adopted in genomics-based studies of regeneration, with publication numbers steadily increasing. While traditional research on organ or somite regeneration remains, it is being progressively supplemented—and in many cases supplanted—by molecular and stem cell–focused studies. Looking forward, continued advances in genomics, stem cell technologies, and regenerative medicine are expected to position planarian research as an important source of theoretical and practical insights for stem cell therapy and regenerative medicine.

### Keywords thematic evolution

**Fig. 5:**
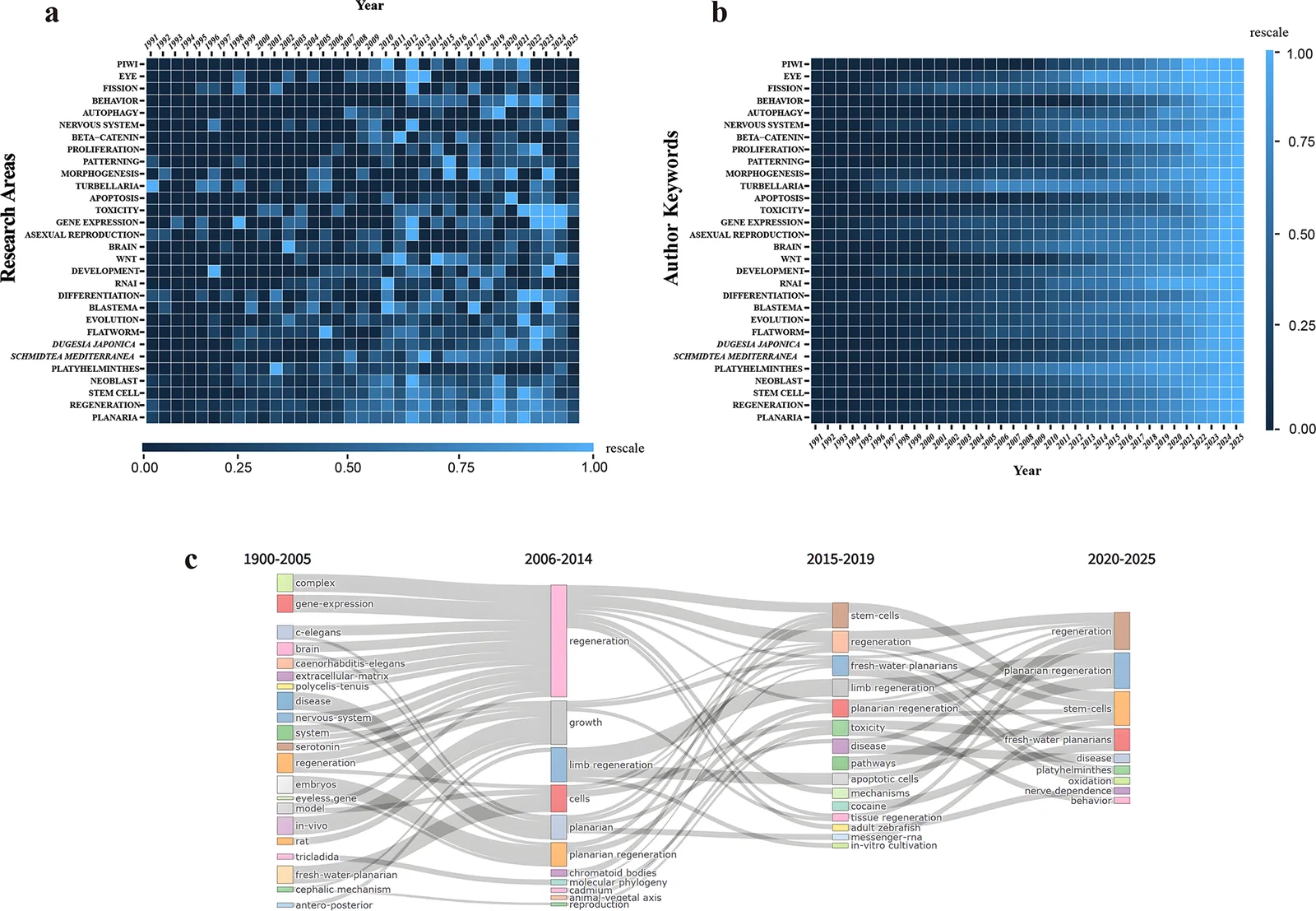
Research areas and thematic evolution. (**a**) Research areas heatmap. This figure shows the popularity of different keywords each year from 1991 to 2025. (**b**) Author keywords heatmap. This figure shows how the research popularity of each keyword changed over time from 1991 to 2025. (**c**) Using the bibliometric package Bibliometrix in the R language environment, the “Thematic Evolution” function under the “Conceptual Structure” toolbar was employed to visualize the evolution of keywords and establish the dynamic relationships of keyword changes across different time periods, spanning from 1900 to 2025. The time segmentation points were established in 2005,2014 and 2019

Based on the horizontal comparison of research area intensity (Fig. 5a), *Turbellaria* emerged earliest as a focal topic. In 1996, a surge in developmental studies highlighted growing interest in the link between flatworms and developmental biology. Around 1998, gene expression became a hotspot, likely driven by advances in molecular techniques such as in situ hybridization, which enabled detailed analyses of gene expression patterns and organismal development [59]. In 2002, research on the planarian brain peaked, alongside related topics such as eye development, nervous system biology, gene expression, and evolution, reflecting the planarian’s value as a model for eye–brain coordination and the evolutionary significance of these organs.

As shown in Figs. 5a and b, 2007 marked the rise of *Schmidtea mediterranea* as a dominant experimental species, attracting more attention than *Dugesia japonica* over the following decade. This shift can be attributed to its diploid stability, in contrast to the mixed ploidy of *D. japonica*, as well as its clearly distinguishable sexual and asexual strains, suitability for comparative studies, and strong regenerative capacity. These traits facilitate the establishment of genetically identical clonal lines from a single individual, minimizing variability and making *S. mediterranea* a high-quality, easy-to-maintain model organism [20, 60].

From 2010 onward, RNA interference (RNAi) gained momentum, as double-stranded RNA–mediated interference became a widely used tool to specifically suppress gene expression, providing critical insights into regeneration mechanisms [19, 61]. In 2012, growing interest in asexual reproduction was linked to fission-based propagation [62]. Between 2010 and 2012, Wnt and β-catenin research expanded rapidly, driven by discoveries showing that the Wnt/β-catenin pathway regulates anterior–posterior polarity in planarians—being activated at the posterior and suppressed at the anterior—thereby providing insights into vertebrate tissue renewal [19, 22, 24, 55].

Research on eye development, which began in 1998, experienced a pause but resurged around 2012, mirroring trends in brain and nervous system studies and reinforcing the developmental relationship between eye and brain [63]. Around the same time, blastema and neoblast research became increasingly prominent, as both are central to regeneration. As Fig. 5b illustrates, regeneration research started relatively late, given its dependence on molecular tools, but interest has remained consistently high [17, 18, 64]. Furthermore, studies of PIWI-like proteins and small RNAs revealed their critical role in regulating stem cell progeny [57].

By 2015, body patterning and morphogenesis had emerged as leading topics, attracting significant attention to body plan regulation and developmental patterning in planarians [24, 65]. Around 2020, apoptosis and morphogenesis jointly became hotspots due to their relevance to regeneration and to key behavioral processes in the planarian life cycle [26, 27, 29]. Following the COVID-19 pandemic, toxicity emerged as a new research focus, while gene expression once again became central, particularly in the context of planarian responses to polluted environments [66, 67].

Keyword evolution analysis (Fig. 5c) shows that research topics in the 20th century were relatively dispersed, with regeneration, gene expression, disease, and in vivo studies as major themes. From 2006 to 2014, regeneration became the dominant focus, building on earlier work while incorporating organ- and cell-level analyses. During this period, limb regeneration has emerged as a significant comparative context for planarian research, with cross-species analyses revealing conserved signaling principles that have attracted substantial academic interest. After 2015, in addition to regeneration, freshwater planarians, and disease, increasing attention has been directed toward stem cells, apoptotic cells, and signaling pathways, reflecting a more mechanistic understanding of regeneration [29, 68]. Concurrently, rising environmental pollution has fueled interest in the ecological impacts of contaminants, especially on freshwater ecosystems. Owing to their exceptional regenerative capacity, freshwater planarians are widely recognized as valuable models for assessing chemical exposure, enabling investigations that range from organismal morphology and behavior to molecular-level mechanisms [66, 69]. Cocaine has also emerged as a focus of planarian research, given its dual role as a surface anesthetic and a widely abused narcotic. Studies on planarians have provided insights into its neurotoxicity and addictive mechanisms, attracting increasing academic attention [70]. In the post-pandemic era, regeneration and stem cells remain central themes, while oxidative stress has become a prominent keyword due to its involvement in degenerative diseases. Research employing planarians to investigate oxidative stress and potential interventions offers promising avenues for understanding pathogenesis and for developing therapeutic strategies [67].

### Analysis of keyword co-occurrence cluster and disciplinary distribution

**Fig. 6:**
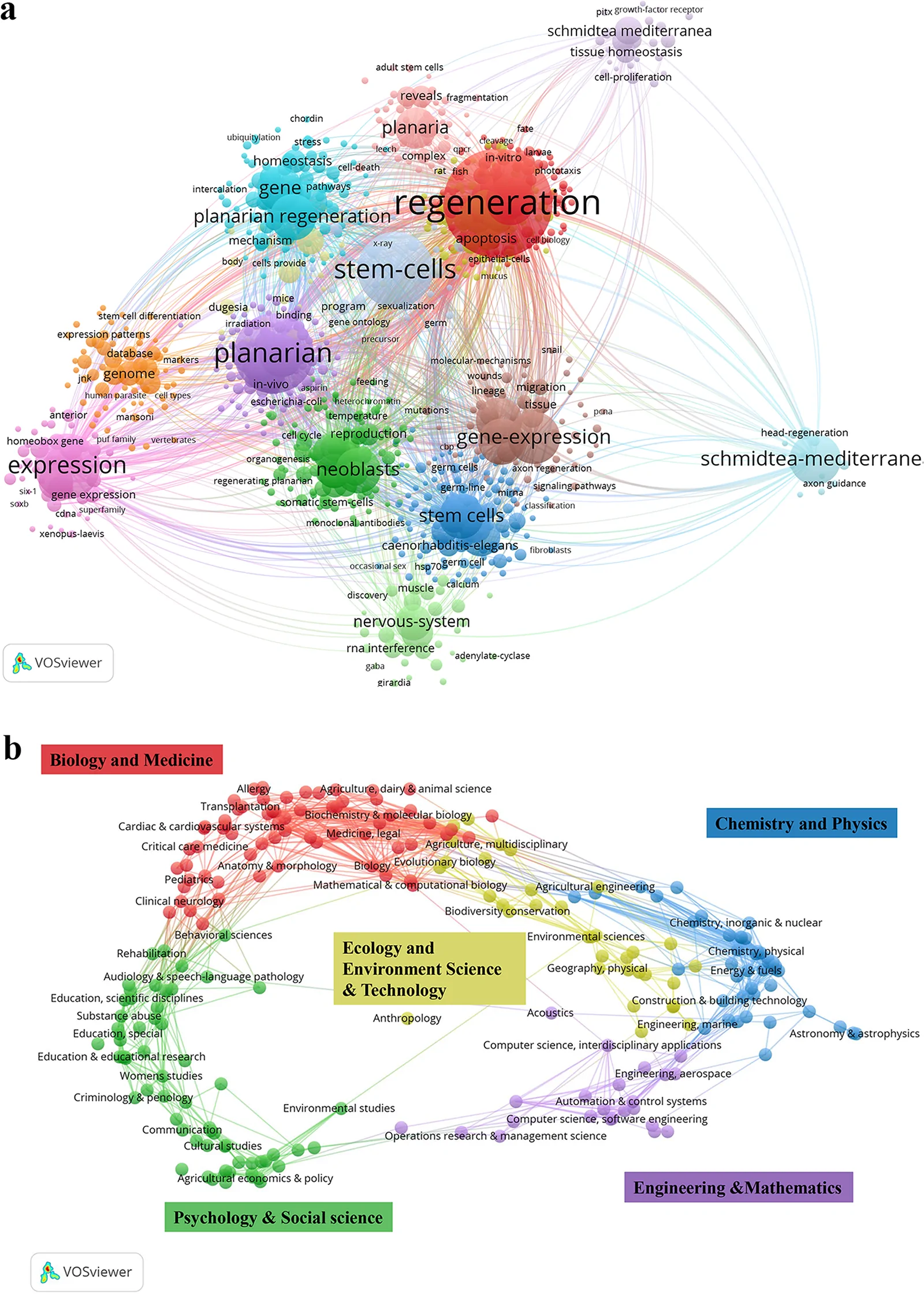
Analysis of keyword co-occurrence cluster and disciplinary distribution. A cluster analysis of the keywords from 1,685 articles was conducted using VOSviewer. Each color represents a different cluster, which consists of multiple closely related terms, and the lines between nodes indicate the strength of co-occurrence among keywords in the analyzed publications. (**b**) Disciplinary distribution analysis. All of the publications are classified into five main subject areas. Different colors represent different disciplines, and the lines connecting different nodes indicate the degree of association between sub-disciplines

Figure 6a depicts the keyword co-occurrence network of planarian regeneration studies (1900–2025), revealing three major sections: (1) core clusters centered on *regeneration*, *stem cells* (gray), and *planarian*; (2) sub-core clusters including *neoblasts*, *stem cells* (blue), *gene expression*, *expression*, and *planarian regeneration*; and (3) peripheral clusters.

The regeneration cluster, occupying a central position, links diverse biological processes and models, emphasizing cellular and molecular mechanisms. Keywords such as *apoptosis* underscore programmed cell death in regeneration, while *epithelial cells* and in vitro suggest the use of epithelial models for studying tissue repair and structural reconstruction. Terms like *phototaxis* and *larvae* broaden the focus to behavior and development, showing that research is not limited to tissue reconstruction but also addresses behavioral responses—such as light avoidance in regeneration-impaired specimens—and organismal maturation [71].

The stem cells cluster associates closely with regeneration, featuring terms like germ, program, and precursor that highlight significant research interest in cell fate determination. The inclusion of methodological terms (X-ray) and bioinformatics frameworks (Gene Ontology) indicates a scholarly shift toward standardized, molecular evaluations of stem cell pathways, rather than relying solely on classical observation [35, 72, 73].

The *planarian* cluster includes in vivo, *irradiation*, *Escherichia coli*, *mice*, and *Dugesia*, illustrating methodological diversity and the widespread application of planarians across molecular, biological, and animal model studies. In contrast, the *planarian regeneration* cluster emphasizes regulatory pathways. *Stress* indicates that damage stimuli and stress responses play a key role in activating regeneration mechanisms. *Chordin* belongs to morphology-related genes and is key to studying axial formation and regeneration localization [26, 74].

The *neoblast* cluster highlights neoblast function and regulation. Keywords such as *cell cycle*, *reproduction*, and *organogenesis* indicate their role in tissue reconstruction and reproductive status, with *temperature* manipulation reflecting environmental control [75]. The inclusion of keywords such as germ-line, miRNA, and hsp70 within the stem cell cluster reflects a significant research trajectory focused on the molecular regulatory layer of neoblast dynamics [76, 77].

The gene-expression, expression and genome clusters feature lineage, homeobox gene, and database, illustrating the integration of transcriptomic technologies into planarian research. The prominence of vertebrate within these clusters underscores a major research trajectory aimed at establishing genetic homology between flatworms and higher vertebrates, which has been elaborated in detail in Table S1 [20, 35].

Finally, the *nervous-system* cluster represents a key domain connected to stem cell and gene-expression studies. Molecular techniques such as RNA interference (*RNAi*) have shifted research from neural control of movement to specialized muscle cells providing positional cues for nerve regeneration [78].

Overall, the dense linkages among these clusters highlight a highly integrated research framework mapping the continuum from organismal biology to molecular regulation. The network demonstrates a profound scholarly focus on the synergistic interplay between stem cell dynamics and spatiotemporal gene control. Crucially, these bibliometric associations represent data-driven macroscopic publication trends rather than subjective future predictions, bridging cellular mechanisms with broader regenerative principles through an objective synthesis of the field’s established scholarly landscape.

Figure 6b categorizes 1,685 publications into five disciplinary clusters: Biology & Medicine (red), Ecology & Environmental Science & Technology (yellow), Chemistry & Physics (blue), Engineering & Mathematics (purple), and Psychology & Social Science (green).

The *red* cluster represents core biological disciplines, covering *biology*, *medicine*, *biochemistry*, *molecular biology*, *allergy*, *anatomy*, *morphology* and *transplantation*—fields fundamental to understanding regenerative mechanisms. At the intersection with the *green* cluster, interdisciplinary studies such as *clinical neurology* and *behavioral sciences* focus on nervous system regeneration (e.g., axonal regrowth and neuronal remodeling) and functional recovery (e.g., phototactic and tactile responses) in planarians [71]. Although planarians lack *auditory* or *language system*, their neural plasticity offers insight into motor and cognitive *rehabilitation*.

The *green* cluster, dominated by *education*, reflects planarians’ role in teaching regeneration and reproduction due to their visibility and minimal ethical concerns [79]. Inclusion of substance abuse demonstrates their utility in addiction studies, intersecting with forensic toxicology and criminology [80].

The *yellow* and *blue* clusters integrate ecology, toxicology, chemistry, and physics. Environmental toxicology studies on pollutants affecting freshwater planarians combine ecological perspectives with physical and chemical assays to assess impacts on regeneration, gene expression, and behavior [67]. Topics such as bioelectricity, oxidative stress, and weak magnetic fields fall under chemistry and physics, yet remain closely tied to planarian biology and their environmental responses [67]. *Agriculture*, *evolutionary biology*, and *biodiversity conservation* at the *red–yellow–blue* interface emphasize planarians’ ecological significance and their role in environmental monitoring and large-scale pollutant screening [80].

The *purple* cluster centers on *engineering*, *automation*, and *computer science*. Computational modeling, ion channel studies, and electric-field manipulation illuminate regulatory algorithms, while genomic databases connect gene expression with macro-level mechanisms, providing a data foundation for interdisciplinary research. Mathematical modeling and simulations predict regeneration patterns and morphogenesis, informing regenerative medicine, synthetic biology, and robotics [81, 82]. *Management science* and *operations research*, at the *green–purple* intersection, reflect the application of experimental resource allocation optimization and resource scheduling, system dynamics modeling, and knowledge flow management in planarian regeneration research. Furthermore, AI-driven simulations and strategic modeling are also used to predict complex behaviors such as stem cell fate evolution and tissue reconstruction [83].

In summary, planarian regeneration and stem cell research, once grounded in biology as well as medicine disciplines, is evolving into a highly interdisciplinary field encompassing environmental science, psychology, chemistry, engineering, and mathematics. In the future, emerging disciplines such as computer science will further promote collaboration between these classical subjects and traditional regeneration mechanism research, establishing a new framework for multi-scale, cross-disciplinary research with greater depth.

### Hotspot evolution analysis

Hotspot Evolution Analysis can reflect research hotspots and developmental trends in the field of planarian stem cell and regeneration studies. Using the Burst Detection function in CiteSpace, we identified the top 20 keywords with the strongest citation bursts from 2000 to 2025 (Table 5).

**Table 5:**
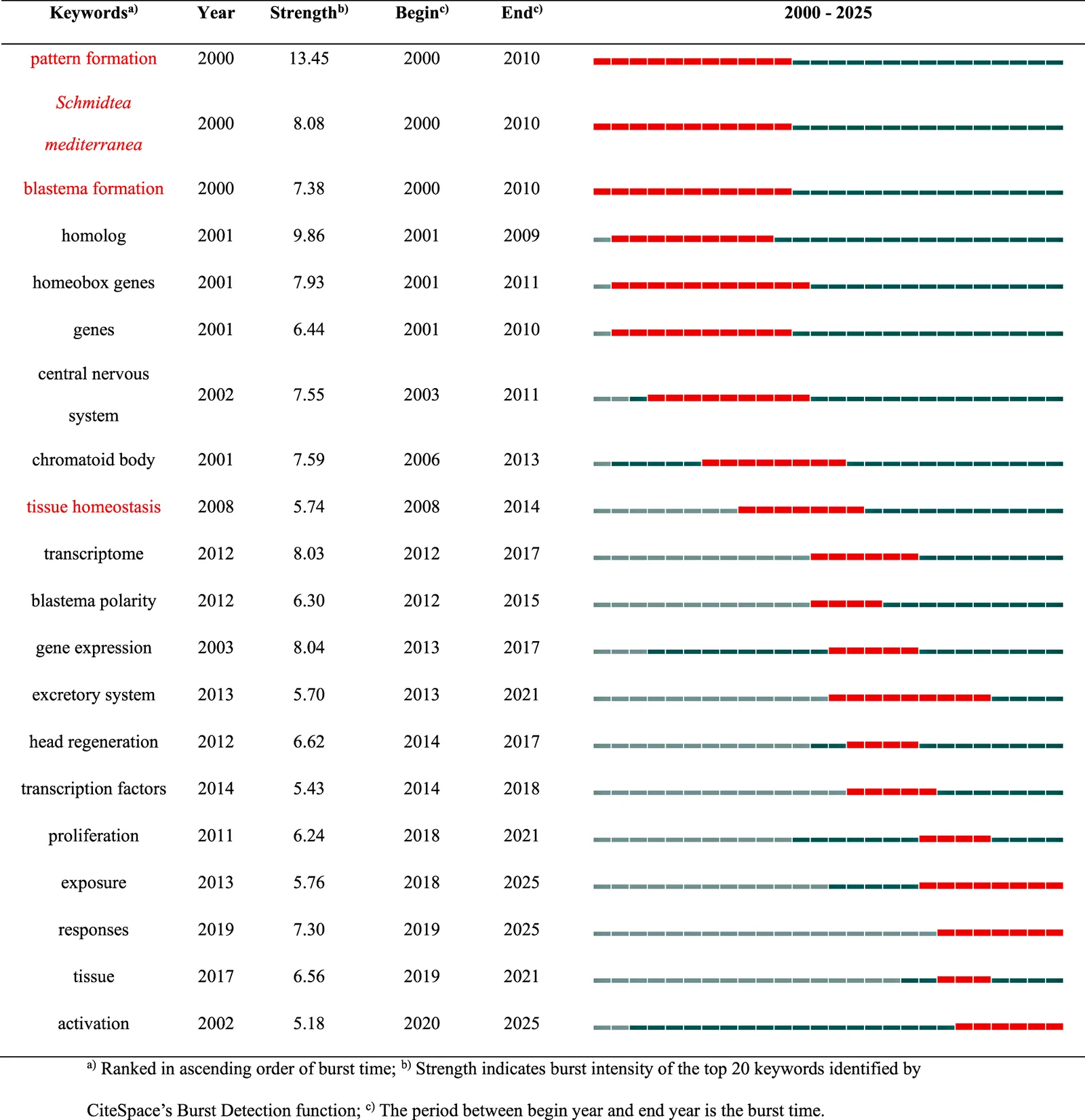
Top 20 keywords with the strongest citation bursts

In the early 2000s, keywords such as *pattern formation*, *Schmidtea mediterranea*, and *blastema formation* exhibited high burst strength, suggesting that research at this stage primarily focused on fundamental mechanisms of morphological reconstruction and blastema formation after injury. *Schmidtea mediterranea* was also established as a representative model organism, laying the theoretical groundwork for subsequent studies on regenerative development. Between 2001 and 2003, terms like *homolog*, *homeobox genes*, *genes*, and *central nervous system* appeared and rapidly gained attention, which implied the increasing recognition of gene regulation—particularly homeobox transcription factors—as central to axial patterning and tissue specification. Concurrently, regeneration of the nervous system became a research frontier, with efforts directed at uncovering general principles of neural tissue organization and repair [84]. From 2006 to 2011, the burst of keywords such as *chromatoid body* and *tissue homeostasis* revealed that research is beginning to delve deeper into the role of cellular structures such as chromatoid body in stem cell function, as well as the regenerative mechanisms that maintain tissue homeostasis. Notably, the identification of chromatoid bodies in neoblasts was considered a landmark of their pluripotency [85, 86]. After 2012, the appearance of *transcriptome*, *gene expression*, and *transcription factors* signaled a clear focus on molecular-level investigations. The development of transcriptome sequencing technologies provided powerful tools for profiling gene dynamics during regeneration, enabling researchers to decipher complex regulatory networks from a systems biology perspective [33, 34]. From 2018 onward, keywords such as *proliferation*, *exposure*, and *responses* became prominent, showing a broadening of scientific inquiry—from endogenous mechanisms to the influence of exogenous stimuli. Topics such as the impact of environmental pollutants on regenerative capacity and the modulation of stem cell proliferation in response to external cues attracted widespread attention. This period also saw increasing integration of environmental science and toxicology into planarian research [80]. In 2020, *activation* became a newly burst keyword, demonstrating a further refinement of studies toward the mechanisms of signal pathway activation during regeneration. Particular attention has been paid to how extracellular signals direct stem cell fate decisions to achieve targeted tissue reconstruction, thereby promoting the conversion of planarians studies into practical applications for regeneration [87]. To contextualize these statistical shifts, Supplementary Table S5, which is adapted from a prior study, summarizes core publications by experimental system and key findings, bridging analytical patterns with the foundational research milestones of the discipline [88].

In aggregate, the evolution of burst keywords suggests a progressive expansion of the field—from early studies on morphological formation and model organism establishment, through gene regulation and stem cell characterization, to recent focus on molecular mechanisms and environmental interactions. This trend not only illustrates the advancement of technological methodologies but also indicates a paradigmatic shift from descriptive studies to mechanistic inquiries and from single-discipline research to interdisciplinary integration.

## Discussion

This study provides a comprehensive bibliometric analysis of the research landscape on planarian stem cells and regeneration, covering more than a century of scientific exploration. By systematically examining publication trends, collaboration networks, keyword clusters, thematic evolution, and disciplinary integration, we have constructed a structured knowledge map that delineates both the historical trajectory and epistemic development of the field, while also emphasizing its increasing interdisciplinarity. Our findings highlight the dynamic shift from descriptive morphological studies to mechanistic molecular research, underscored by the pivotal role of neoblasts and signaling pathways in regeneration, alongside growing integration with environmental science, computational biology, and toxicology.

The temporal analysis of publications reveals three distinct phases of growth, each reflecting broader technological and conceptual transitions in the life sciences. The initial slow progression (1900–2010) was dominated by morphological and observational studies, constrained by the absence of advanced molecular tools. The subsequent phase of rapid expansion (2011–2020) coincided with the rise of high-throughput sequencing and functional genomics, which facilitated deeper insights into stem cell pluripotency and genetic regulation [19, 22]. The most recent phase (2021–2025) shows a relative decline, likely influenced by pandemic-related disruptions, but also indicative of field maturation and thematic specialization. These trajectories parallel the broader development of regenerative biology, where planarians have established themselves as a central model for investigating conserved mechanisms of tissue renewal and stem cell biology [56, 89].

Our co-authorship and institutional analyses highlight the dominant role of the United States and European nations in driving research output and collaboration, with China emerging as a significant yet comparatively isolated contributor. This asymmetry may reflect disparities in research investment, historical engagement, or access to collaborative networks. The prominence of institutions such as Harvard University and the University of Barcelona underscores the importance of established research ecosystems in sustaining long-term investigations. These findings align with global patterns in model organism research, where resource-rich institutions frequently lead the generation of high-impact data and shared resources such as genomic databases [56, 90].

Keyword co-occurrence and burst-detection analyses further reveal a clear conceptual transition from organ-level regeneration to cellular and molecular mechanisms. Whereas early studies emphasized blastema formation and tissue patterning, more recent work has focused on gene regulation, transcriptomics, and environmental interactions. The sustained prominence of terms such as *neoblast*, *Wnt signaling*, and *β-catenin* underscores their central role in axial patterning and stem cell regulation [22, 23]. In contrast, the emergence of themes such as *toxicity*, *apoptosis*, and *oxidative stress* highlights a growing emphasis on environmental and translational applications. This trajectory is consistent with the broader rise of ecotoxicology and chemical screening [66, 67]. where planarians are increasingly employed as scalable and ethically favorable models for high-throughput assays [81].

The clustering of keywords and the disciplinary distribution map further highlight the remarkable convergence of previously distinct fields, positioning planarian research at the intersection of developmental biology, environmental science, computational modeling, and even the social sciences. This interdisciplinary integration is not only thematic but also methodological and conceptual, reflecting a broader shift in contemporary science toward systems-level understanding. For example, the application of planarians in environmental toxicology—particularly in evaluating the effects of emerging contaminants such as microplastics, pesticides, and pharmaceuticals—demonstrates their utility as bioindicators in ecotoxicological studies [91, 92]. Their sensitivity to chemical exposure, combined with their regenerative capacity, provides a unique model for investigating long-term sublethal effects and recovery mechanisms, which are often difficult to capture in vertebrate models.

Similarly, the intersection with neuroscience and behavioral science has extended the scope of planarian research beyond regeneration into neuropharmacology and addiction studies. The resurgence of keywords such as *cocaine* and *behavior* reflects a growing interest in employing planarians to model substance abuse and neurotoxicity, capitalizing on their simple yet functional nervous system and high-throughput compatibility [80]. This approach not only provides insights into conserved mechanisms of drug action but also aligns with the 3Rs principles (Replacement, Reduction, Refinement) in toxicological testing.

In education and public engagement, planarians have emerged as a valuable tool for teaching fundamental concepts in stem cell biology, regeneration, and experimental design. Their ease of maintenance, rapid regenerative ability, and ethical acceptability make them well suited for use in primary and secondary schools as well as undergraduate curricula, thereby fostering early scientific literacy and inquiry-based learning [79]. From a methodological perspective, the integration of computational and engineering approaches—such as agent-based modeling, machine learning, and bioelectric mapping—has begun to reveal principles of self-organization and pattern formation that extend beyond planarians. Computational models of planarian regeneration, for example, have guided the development of adaptive systems in robotics and synthetic biology [81, 82], underscoring the role of planarians as a “model model” for reverse-engineering complex biological processes.

Looking ahead, several promising research directions may further enhance the scientific and translational impact of planarian studies:

(i). The application of spatially resolved transcriptomics and live imaging technologies could provide unprecedented resolution of neoblast dynamics and lineage tracing in real time, illuminating how spatial cues and cellular interactions orchestrate regeneration. Coupled with CRISPR-based functional screens, these approaches may identify novel regulators of stemness and differentiation.
(ii). The exploration of bioelectric signaling in planarian regeneration warrants deeper investigation. Preliminary evidence suggests that ion channels and electrical gradients play critical roles in patterning and repair [93], yet a systematic integration of biophysical and molecular approaches is needed to clarify the underlying mechanisms.
(iii). The role of planarians in drug discovery and toxicology should be expanded through standardized assays and automated platforms. Their suitability for high-throughput screening could accelerate the identification of pro-regenerative compounds and environmental toxicants, contributing to both regenerative medicine and environmental health policies.
(iv). The development of planarian-specific databases and analytical tools—such as single-cell atlases, gene regulatory networks, and phenomic resources—would facilitate data sharing and collaborative research across disciplines. Initiatives like PlanMine [36] represent important progress, but further integration with multi-omic datasets and computational resources is essential.
(v). Finally, the ethical and philosophical implications of planarian research—particularly regarding consciousness, nociception, and regeneration—deserve broader discussion. As planarians are increasingly utilized in behavioral and neurotoxicological studies, the establishment of ethical guidelines and interdisciplinary dialogue will be critical.

Alternative explanations for the observed publication trends should be considered. First, the increase in molecular and transcriptomic studies after 2010 coincides with the general decline in sequencing costs and the widespread adoption of high‑throughput technologies across all model organisms. Therefore, the shift toward molecular mechanisms may reflect broader technological advances rather than field‑specific intellectual evolution. Second, the emergence of environmental toxicology as a research theme after 2018 may partly result from increased research funding for pollution monitoring and ecotoxicology, rather than a unique suitability of planarians for such studies. Third, the relative decline in publication output after 2020 could be attributed to COVID‑19‑related laboratory disruptions, which affected many experimental fields, rather than a genuine loss of scientific interest. These alternative interpretations do not invalidate our findings but highlight the importance of contextualizing bibliometric trends within broader scientific and socioeconomic factors.

A legitimate question is whether the trends we observed—particularly the transition from descriptive morphology to molecular mechanisms—simply mirror the general trajectory of life sciences rather than representing field-specific characteristics. To address this, we compared our keyword trends with broader biological literature patterns reported in previous bibliometric studies [94]. While the shift toward molecular biology is indeed a general phenomenon, several aspects appear more specific to planarian research: (1) The sustained centrality of “neoblasts” and “blastema formation” reflects the unique biology of planarian stem cells. (2) The prominence of “body axis patterning” via Wnt/β-catenin signaling, while also studied in other models, has a particularly well-defined role in planarian anterior-posterior specification. (3) The recent integration with “environmental toxicology” is less pronounced in classical model organisms such as *Drosophila* or *C. elegans*. Therefore, while general technological advances have certainly shaped the field, planarian research has maintained distinct thematic characteristics that justify its separate analysis.

### Limitations of the study

This study has several limitations inherent to bibliometric methodology. First, our analysis relies exclusively on the Web of Science Core Collection (WoSCC). While WoSCC is a rigorous and curated database for high-impact literature, it may underrepresent publications from regional journals or non-English sources, potentially introducing a geographical or linguistic bias. Second, despite rigorous keyword cleansing, the initial search strategy is limited by the specificity of the query terms. Studies focusing on planarian regeneration but employing alternative or highly specialized nomenclature (e.g., specific gene names as title terms without mentioning the organism context) might have been excluded. Furthermore, we intentionally included studies involving non-planarian regenerative models, as these cross-species analyses are essential for contextualizing conserved regenerative principles within the broader scientific landscape. Third, bibliometric co-occurrence networks identify statistical associations between terms, not direct biological causation. Therefore, while the clusters (Fig. 6a) highlight conceptual proximity in the literature, they should be interpreted as maps of research activity rather than functional or mechanistic pathways per se. Finally, citation‑based metrics, while widely used in bibliometric studies, have well‑documented limitations including preferential citation of older papers, field‑dependent citation practices, and the influence of review articles over original research. These metrics reflect scientific visibility and attention rather than direct measures of research quality or innovation. In our study, citation metrics inherently favor older publications and established groups, which may obscure recent, high‑quality contributions that have not yet accumulated significant citation counts. Our interpretations therefore focus on macroscopic patterns of research activity rather than making claims about the relative importance of individual contributions and scientific quality.

## Conclusion

In conclusion, this bibliometric analysis highlights the dynamic and increasingly interdisciplinary nature of planarian stem cell and regeneration research. By synthesizing decades of scholarly output into a coherent knowledge framework, we provide a foundation for future studies aiming to bridge fundamental biology with applied science. As the field embraces emerging technologies, such as spatially resolved transcriptomics, whole-body 3D imaging, CRISPR-based functional screening, and computational modeling, planarians represents a valuable model for generating testable hypotheses in regeneration and stem cell biology. While bibliometric data reveal the structure and evolution of the field, future experimental validation remains essential to translate these mapped trends into mechanistic and therapeutic insights.

## Supplementary Information

Below is the link to the electronic supplementary material.

Supplementary Material 1

Supplementary Material 2

## Data Availability

Data and materials will be made available on request.

## Abbreviations

SCIE: Science Citation Index Expanded
SSCI: The Social Sciences Citation Index
WoSCC: The Web of Science Core Collection
