# Supplementary material for: Mapping the knowledge landscape of planarian regeneration: a century of bibliometric insights
Source: Biol Direct. 2026 Jul 17;21:200. doi: 10.1186/s13062-026-00906-0 (PMC13659995; doi:10.1186/s13062-026-00906-0)
Supplement: Supplementary file 1 [file 13062_2026_906_MOESM1_ESM.doc]

**Mapping the Knowledge Landscape of Planarian Regeneration: A Century of Bibliometric Insights**

**Xinyang Wu****^1,2,3^**^†^**, Fang Zhan^1,2^****^,3^**^†^**, Xiaoran Zhang^1,2,3^**^†^**,** **Haoyang Zhang^1,2,3^, Ya Wang^1,2,3^ and Ting Wang^1,2,3*^**

^1^ National Demonstration Center for Experimental Basic Medical Education, School of Basic Medicine, Tongji Medical College, Huazhong University of Science and Technology, Wuhan, Hubei 430030, China

^2^ Department of Pathogen Biology, School of Basic Medicine, Tongji Medical College, Huazhong University of Science and Technology, Wuhan, Hubei, 430030, China

^3^ Innovation Research Center, School of Basic Medicine, Tongji Medical College, Huazhong University of Science and Technology, Wuhan, Hubei 430030, China

* Address correspondence to: Ting Wang; [wangting139@hust.edu.cn](mailto:wangting139@hust.edu.cn)

† These authors have contributed equally to this work and share first authorship.

Contents

**Supplementary Table S1 |** Evolutionary Conservation and Domain-Specific Identity of Key Planarian Regeneration Genes

**Supplementary Table S2 |** Study selection criteria (PRISMA-based)

**Supplementary Table S3 |** Keyword standardization

**Supplementary Table S4 |** Annual Growth Rate of Publications and 5-year Moving Average Growth Rate of Publications (supplied as a separate Excel Table S4 file)

**Supplementary Table S5 |** Summary of planarian regeneration literature by cluster, species, methods, and phenomena

**The full terms of the abbreviations used in the Table S5**

**Supplementary Figure S1 |** 5-year Moving Average Growth Rate of Publications (From 2000 to 2025)

**Reference**

Supplementary Table S1 | Evolutionary Conservation and Domain-Specific Identity of Key Planarian Regeneration Genes

| **Planarian Gene (S. med) ^a)^** | **Mammalian Orthologue (Human) ^b)^** | **Target Functional Domain ^c)^** | **Domain Identity (%) ^d)^** | **Biological Function in Regeneration** | **References** |
| --- | --- | --- | --- | --- | --- |
| *soxB1* | *SOX2* | HMG box | ~88% | CNS neuronal maintenance and sensory neurogenesis. | [1] |
| *lhx1/5-1* | *LHX1* | Homeobox domain | ~85% | Brain patterning and neural network reconstruction. | [2] |
| *pitx* | *PITX2* | Homeodomain | ~78% | Specification of serotonergic neurons in the CNS. | [3] |
| *hippo* | *MST1* | Protein kinase domain | ~76% | Organ scaling via cell cycle and apoptosis control. | [4] |
| *bmp4* | *BMP4* | TGF-β C-terminal domain | ~74% | Dorsal-ventral (D/V) axis specification. | [5] |
| *myoD* | *MYOD1* | bHLH domain | ~65% | Master regulator for muscle fiber formation. | [6] |
| *wnt1* | *WNT1* | Wnt domain (Cys-rich) | ~52% | Anterior-posterior (A/P) axis patterning. | [7] |
| *egfr-3* | *EGFR* | Protein kinase domain | ~48% | Neoblast self-renewal and fate determination. | [8] |
| *slit* | *SLIT2* | LRR (Leucine-rich repeat) | ~45% | Midline maintenance and axon guidance. | [9] |
| *ovo* | *OVOL2* | C2H2-type zinc finger | ~45% | Photoreceptor progenitor differentiation. | [10] |
| ^a)^ Genes are listed using standard nomenclature (lowercase italics) and arranged in descending order based on their domain identity percentages.  ^b)^ A single representative human orthologue was selected for each gene family, prioritizing the highest sequence identity and the most conserved functional role in regeneration or development.  ^c)^ Conserved domains (e.g., HMG box, Homeobox) were identified using Pfam and InterPro databases to ensure functional relevance.  ^d)^ Values indicate the percentage of amino acid identity within the specified functional domain, calculated via NCBI BLASTp tool [11]. | | | | | |

**Supplementary Table S2 | Study selection criteria (PRISMA-based) ^a)^**

| **Criterion** | **Inclusion** | **Exclusion** |
| --- | --- | --- |
| Database | Web of Science Core Collection (SCIE) | Other databases |
| Time span | January 1, 1900 – June 17, 2025 | Outside this range |
| Document type | Article; Review | Conference abstract; Editorial; Letter; Correction; Book chapter; News item; Withdrawn publication; Reprint |
| Language | English | Non-English (e.g., Chinese, German, etc.) |
| Relevance (manual) | Directly related to planarian regeneration | Irrelevant topics |
| Duplicates | Removed using CiteSpace | — |
| a) This table follows the PRISMA (Preferred Reporting Items for Systematic Reviews and Meta Analyses) framework. The initial search yielded 1,956 records. After applying the exclusion criteria (document type, language, and manual screening), 1,685 publications were retained for bibliometric analysis. No additional records were excluded due to low relevance or duplication beyond the automated removal of duplicates by CiteSpace. | | |

**^Supplementary Table S3 | Keyword standardization^**a)

**Original keyword variants**

| **Standardized keyword** |  |
| --- | --- |
| activation; signaling activation; pathway activation; transcriptional activation; upregulation | activation |
| Blastema; blastema formation; blastemal; blastemal cells | blastema formation |
| Cell proliferation; proliferation; proliferative | proliferation |
| central nervous system; CNS | central nervous system |
| chromatoid body; chromatoid body-like structure | chromatoid body |
| Differentiation; cell differentiation | differentiation |
| *Dugesia japonica*; *Dugesia*; *D. japonica* | *Dugesia japonica* |
| excretory system; excretory network | excretory system |
| exposure; chemical exposure; toxicant exposure; stress exposure | exposure |
| Gene expression; gene expression profiling; gene expression analysis | gene expression |
| genes; gene | genes |
| *Girardia tigrina*; *Girardia*; *G. tigrina* | *Girardia tigrina* |
| head regeneration; anterior regeneration; head regrowth; cephalic regeneration; head formation | head regeneration |
| homeobox genes; Hox genes; homeodomain genes | homeobox genes |
| homolog; homologue; sequence homolog; conserved homolog | homolog |
| In situ hybridization; ISH | in situ hybridization |
| Neoblast; Neoblasts; neoblast stem cell; neoblast cells | neoblasts |
| Pattern formation; patterning; axial patterning | pattern formation |
| Planarian; Planarians; Planaria; freshwater planarians | planaria/planarian |
| Positional information; positional cues | positional information |
| Progenitor cell; Progenitor cells; Mother cell; Mother cells | progenitor cell |
| Regeneration; Regenerations; Endogenous regeneration; Repair | regeneration |
| responses; stress response; cellular response; regeneration response; wound response | responses |
| RNA interference; RNAi | RNAi |
| *Schmidtea mediterranea*; *Smed*; *S. mediterranea* | *Schmidtea mediterranea* |
| Single cell sequencing; scRNA-seq; single-cell RNA-seq; single cell RNA sequencing | single‑cell RNA‑seq |
| Stem cell; Stem cells; Adult stem cell; Adult stem cells; Somatic stem cell; Somatic stem cells | stem cells |
| Tissue homeostasis; homeostasis | tissue homeostasis |
| tissue; body tissue; organ tissue; differentiated tissue; tissue type | tissue |
| transcription factors; TFs; transcriptional regulators; DNA-binding transcription factors | transcription factors |
| transcriptome; transcriptome profiling | transcriptome |

^a)^ This table provides the standardized terms for this paper. The consolidation was performed independently by two authors and cross-verified. Singular/plural forms and synonymous terms were merged into unified keywords as shown above.

**^Supplementary Table S5 | Summary of planarian regeneration literature by cluster, species, methods, and phenomenaa)^**

| **Major topic** | **Target species** | **Methods/Approaches** | **Biological phenomena** | **Key cited references** |
| --- | --- | --- | --- | --- |
| **1. Morphogenesis & blastema formation** | *Dugesia japonica* | cDNA^b^ Clones,RNAi (dsRNA injection), whole‑mount ISH and immunohistochemistry, RT‑qPCR, X‑ray irradiation | Hedgehog signaling regulates AP polarity: *Djptc* RNAi → Janus‑tails (bipolar tails); *Djhh* or *Djgli* RNAi → Janus‑heads (bipolar heads). Hh acts upstream of Wnt, inducing *DjwntP‑1* in differentiated neural cells along VNCs to specify posterior fate. | [12] |
|  | *Schmidtea mediterranea* | RNAi (myoD, nkx1‑1, double), FISH, immunostaining, TEM, amputation, mRNA‑seq | myoD → longitudinal fibers → regeneration initiation; nkx1‑1 → circular fibers → ML patterning; double → homeostatic defects | [6] |
|  | *Schmidtea mediterranea* | Transplantation (pole/flank fragments), RNAi (wnt5, slit, bmp4, smad1), FISH, BrdU pulse‑chase, irradiation, amputation (transverse/oblique/sagittal/DV‑oblique) | Anterior pole (foxD⁺/notum⁺/zic‑1⁺) forms at intersection of three landmarks: anterior‑facing wound, pre‑existing midline (slit⁺), and DV boundary (laminB⁺). Pole progenitors specified at old midline (ML) but broadly along DV axis, then coalesce at blastema DV boundary. Wnt5/Slit regulate ML width; Bmp signaling positions pole on DV axis. Pole acts as organizer for head pattern (AP & ML). | [13] |
| **2. Gene regulation & patterning** | *Dugesia japonica* | RNAi (soaking), WISH, FISH, immunostaining (H3P, SYNAPSIN), flow cytometry (Hoechst/Calcein/PI), TUNEL, qRT‑PCR, RNA‑seq, X‑ray irradiation, amputation‑regeneration assay | DjWTAP (Wilms’ tumor 1‑associating protein) widely expressed in neoblasts and parenchyma; RNAi causes head involution, erosion, death within 20 days, blocks regeneration (no head/tail). Increases neoblast proliferation (PiwiA⁺, H3P⁺, X1 cells) but impairs differentiation of epidermal (NB.21, Agat‑1), neural (Chat, Synapsin), intestinal (Gata1/2/3) and excretory lineages. Upregulates TRAF6; double RNAi (DjWTAP + DjTRAF6) rescues homeostasis and regeneration. | [14] |
|  | *Schmidtea mediterranea* | RNAi, FISH/WISH, Ribo‑seq, RiboMeth‑seq, immunostaining, TEM | fbl‑1 → neoblast proliferation & early progenitor differentiation; fbl‑2 → late epidermal specification; both control rRNA 2'‑O‑methylation & translation of specific mRNAs (splicing, Wnt signaling). KD impairs regeneration & tissue homeostasis. | [15] |
|  | *Dugesia japonica* | RNAi, FoxO inhibitor (AS1842856), WISH/FISH, immunostaining (H3P, Ac‑tubulin, SYNORF1), TUNEL, Western blot (p‑Erk), qRT‑PCR, RNA‑seq, amputation | FoxO required for neoblast differentiation (not proliferation) via Erk signaling; knockdown blocks regeneration of CNS, eyes, gut, pharynx, reduces lineage progenitors, impairs cilia & mobility. | [16] |
| **3. Stem cell properties & tissue homeostasis** | *Schmidtea mediterranea*, *Dugesia japonica*, *Girardia tigrina* | RNAi, whole‑mount ISH, immunostaining (anti‑synapsin, anti‑VC1), tissue transplantation, gap‑junction blockers (heptanol/hexanol), amputation‑regeneration assay | Wnt/β‑catenin forms posterior‑high gradient, controls AP identity (graded knockdown produces tailless → two‑headed → radial hypercephalized). BMP pathway (BMP, Smad1, Smad4) patterns DV axis; silencing causes ventralization or Siamese twins. *nou‑darake* (FGF receptor‑like) restricts brain to head. Posterior Wnts (wntP‑1, wnt11‑2) act as morphogens. Gap junctions required for axial patterning. Default state of neoblasts is head formation. | [17] |
|  | *Dugesia japonica*; *Phagocata kawakatsui* | RNAi (injection), MEK inhibitor (U0126), WISH, immunostaining (pERK, anti‑AADC, anti‑SYT, anti‑arrestin), qRT‑PCR, behavioural assay (phototaxis), amputation‑regeneration assay | ERK signaling forms anterior gradient; Wnt/β‑catenin posterior gradient. *nou‑darake* (FGF receptor‑like) restricts ERK to head; β‑catenin suppresses ERK outside head. Loss of β‑catenin or *ndk* causes anteriorization (ectopic head/eyes). ERK acts as default “head stuff”. In *P. kawakatsui*, high β‑catenin blocks head regeneration from tail; β‑catenin RNAi rescues head formation. | [18] |
|  | *Schmidtea mediterranea* | RNAi (feeding), WISH, FISH, immunostaining (ARRESTIN, BrdU), light avoidance assay, organ resection (eye/pharynx), BrdU pulse‑chase, axis truncation/remodeling | notum RNAi → anterior ectopic eyes (regenerative) + pre‑existing posterior eyes (non‑regenerative but homeostatically maintained). wnt11‑6; fzd5/8‑4 RNAi → posterior ectopic eyes (regenerative). wntP‑2; ptk7 RNAi → posterior ectopic pharynx (regenerative). Pre‑existing and ectopic organs compete for migratory progenitors; total eye cell number unchanged. Severe axis truncation naturally shifts eye regeneration site. Regeneration and homeostasis can be uncoupled. | [19] |
| **4. Transcriptomics & molecular networks** | *Schmidtea mediterranea* | RNAi (dsRNA injection), whole‑mount ISH, immunostaining (anti‑synapsin, anti‑α‑tubulin, anti‑actin, anti‑arrestin, anti‑NPF), confocal microscopy, SEM, TEM, motility analysis, amputation‑regeneration assay | Smed‑dvl‑1/2 (Dishevelled) functions in three pathways: (1) canonical Wnt/β‑catenin → AP axis (double RNAi causes radial hypercephalized, loss of trunk/tail). (2) β‑catenin‑independent via Wnt5 → neural connectivity, mediolateral CNS patterning, visual axon guidance (disconnected/displaced brains, aberrant optic chiasm). (3) Planar cell polarity (PCP) via Dvl‑2, Vang‑1/2, Div → apical docking of ciliary basal bodies, actin organization, ciliogenesis (defective cilia, loss of polarity, “inching” movement). | [20] |
|  | *Schmidtea mediterranea* | Integrated transcriptomics (RNA‑seq from 5 experiments), genome‑scale metabolic reconstruction, Pfam/InterPro annotation, transcription factor prediction (PhyloPro), cadherin phylogeny, transposon analysis (RepeatMasker), Drop‑seq single‑cell RNA‑seq (~2000 cells), Seurat/PAGODA clustering, in situ hybridization validation | Transcriptome defines 35,232 high‑confidence transcripts; expanded gene families: DDE transposons, TRAFs (MATH domain), cadherins (94). scRNA‑seq resolves 11 cell clusters (neoblast, muscle, neural, gut, epithelial, parapharyngeal, cathepsin⁺ mesenchymal, pigment cells, transient progenitors). Metabolic reconstruction reveals pathways lost in parasitic flatworms (fatty acid, branched‑chain amino acid degradation, mucin‑type O‑glycan). Transcription factor analysis identifies 843 TFs, including egr‑5 in optic cup regeneration. | [21] |
|  | *Schmidtea mediterranea* (asexual strain BCN10) | RNA‑seq (SOLiD 4HT), FACS (X1/X2/Xin), irradiation (60 Gy), RNAi (dsRNA injection), WISH, FISH, immunohistochemistry (anti‑PIWI1), qRT‑PCR, amputation‑regeneration assay | 44 blastema transcription factors (31 novel) enriched in anterior regenerating blastema. Knockdown of 13 (e.g., *Smad4*, *Lmx1a*, *Egr1*, *Zfp*, *MyoD*, *Tbx2/3*, *Traf5*, *Etv6*) causes defects in head organizer, muscle patterning, brain, eye, intestine, and tail regeneration. Many TFs co‑expressed with tissue markers (PC2, myhc, porcn1, gH4) and PIWI1⁺ progeny cells. | [22] |
| **5. Exogenous stimuli & environmental toxicology** | *Schmidtea mediterranea* | RNAi (wtap), MeRIP‑seq, RNA‑seq, scRNA‑seq, UHPLC‑MRM‑MS/MS (m⁶A quantification), BrdU labeling, TUNEL, whole‑mount FISH, immunofluorescence (H3P, 6G10, Ac‑tubulin, GRN), western blot, qRT‑PCR, amputation‑regeneration assay | m⁶A modification essential for regeneration; wtap (m⁶A methyltransferase regulatory subunit) knockdown → abolished regeneration (no head/tail, smaller blastema), disrupted CNS, excretory system, muscle, increased apoptosis, altered cell cycle (↑cdk7/cdk9). scRNA‑seq identifies a unique neural progenitor‑like (NP‑like) cell cluster expressing gm (granulin), whose increased expression upon wtap KD inhibits regeneration; double KD of wtap with gm/cdk7/cdk9 partially rescues regeneration. | [23] |
|  | *Dugesia japonica* | Acute toxicity test (96‑h LC₅₀), enzyme assays (T‑SOD, CAT, GPx, GST, T‑AOC, MDA), morphology & histology (H&E), whole‑mount immunofluorescence (anti‑H3P), qRT‑PCR (piwiA, prog1, agat1, cyclinA/B, Caspase3/8), chromatin dispersion test (DNA fragmentation), TUNEL apoptosis assay, growth & regeneration measurement (body area, blastema size) | MC‑LR (microcystin‑leucine arginine) exposure causes time‑/dose‑dependent mortality, tissue damage (epidermis loss, parenchyma cavities, pharynx regression), degrowth (body area reduction), delayed regeneration (smaller blastema), pigment anomalies (albinism). Induces oxidative stress (altered antioxidants, increased MDA), DNA damage, reduced neoblast proliferation/differentiation (↓H3P⁺, ↓piwiA/prog1/agat1/cyclinB), and increased apoptosis (↑TUNEL, ↑Caspase3/8). | [24] |
|  | *Dugesia japonica* | Acute toxicity (LC₅₀), phenotypic & locomotor tracking (UMATracker/ToxTrac), SEM (cilia), histology (Cason's stain), oxidative stress assays (ROS, SOD, CAT, GPX, GST, MDA), chromatin dispersion test (DNA damage), qRT‑PCR (piwiA, pcnA, cyclinA/B, prog1, agat1, prss12, pou2/3, CAVII, inx10, LC1, α‑tubulin, myoD, collagen, MHCB, pc2, caspase‑3/8, hsp70, MnSOD, CuZnSOD, gst, gpx, nak), Western blot (H3P, 3C11, Cleaved Caspase‑3), whole‑mount immunofluorescence (H3P, SYNAPSIN), TUNEL, amputation‑regeneration assay | DBP (dibutyl phthalate) exposure causes dose‑dependent lethality, morphological abnormalities (shrinking, histolysis), locomotor deficits (reduced speed/acceleration), cilia damage (thickened, shortened, less dense), muscle and intestinal damage, delayed regeneration (smaller blastema, delayed head/tail outgrowth), neurodevelopmental defects (smaller, malformed CNS, reduced pc2). Suppresses stem cell proliferation (↓H3P⁺, ↓piwiA/pcnA/cyclinB) and differentiation (↓prog1/agat1/prss12, ↓muscle/cilia/protonephridia markers). Induces oxidative stress (↑ROS, altered antioxidants, ↑MDA), DNA damage (chromatin dispersion), and apoptosis (↑TUNEL, ↑caspase‑3/8, ↑Cleaved Caspase‑3). | [25] |
| **6. Signaling activation & translational applications** | *Schmidtea mediterranea* | RNAi (feeding dsRNA), WISH, FACS (X1/X2/Xins), whole‑mount immunofluorescence (H3P, caspase‑3, RAD51, γH2AX, synapsin), COMET assay, karyotyping, western blot, qRT‑PCR, amputation‑regeneration assay | Ubc9 (SUMO E2 conjugating enzyme) required for tissue homeostasis & regeneration; knockdown causes posterior‑specific tail loss, reduced neoblast proliferation (↓H3P⁺, ↓X1 cells), G1 arrest, increased DNA damage (DSBs, chromosomal aberrations), defective RAD51 nuclear translocation, and regional apoptosis (↑caspase‑3, ↑Annexin V). Dysregulates Hedgehog pathway (↑Ptc, fluctuating Hh, altered sfrp1/fz4). Regeneration impaired: no posterior blastema, smaller brain, truncated nerve cords. | [26] |
|  | *Schmidtea mediterranea* | Laser‑capture microdissection (LCM), RNA‑seq, whole‑mount ISH, FISH, immunofluorescence (6G10), RNAi (dsRNA feeding), qRT‑PCR, bioinformatics (GO, Human Protein Atlas, scRNA‑seq comparison), mucin domain search | Intestinal transcriptome: 1,844 intestine‑enriched genes; three cell types (phagocytes, goblet cells, basal cells) with medial/lateral subpopulations; regionalized gene expression along mediolateral axis. Functional conservation: homologs enriched in human digestive tissues (duodenum, small intestine, liver). Goblet cell regulation: gli‑1 required for differentiation in regenerating branches; RREB2 required for maintenance in homeostatic intestine; knockdown causes feeding failure and lethality. | [27] |
|  | *Dugesia japonica* | RNAi (dsRNA soaking), WISH, FISH, qRT‑PCR, whole‑mount immunofluorescence (anti‑H3P, anti‑SYNORF1), NOS activity & NO content assays, light sensitivity (negative phototaxis) assay, amputation‑regeneration assay | DjCapon (CAPON homolog) expressed in neural tissue; knockdown impairs neural regeneration (delayed brain lateral branches, reduced GABAergic neurons), decreases NOS activity and NO levels, reduces light sensitivity. Upregulates Wnt/Ca²⁺ pathway genes *DjWnt5a*, *DjCaln*, *DjCamKII* (but not *DjPLCβ*). No effect on blastema size or stem cell proliferation (H3P, piwiA). | [28] |

^a)^ Summary of a representative study on planarian intestine transcriptomics and regeneration. Methods include LCM, RNA‑seq, ISH, RNAi; key findings: 1,844 intestine‑enriched genes, three cell types with mediolateral subpopulations, gli‑1 required for goblet cell regeneration, RREB2 for maintenance. Format follows that of Imperadore and Fiorito [29].

**The full terms of the abbreviations used in the Table S5 are as follows:**

AADC, aromatic L‑amino acid decarboxylase; AP, anteroposterior; BMP, bone morphogenetic protein; BrdU, bromodeoxyuridine; CAT, catalase; CAVII, carbonic anhydrase VII; Chat, choline acetyltransferase; cDNA, complementary DNA; CNS, central nervous system; COMET, COMET assay; CuZnSOD, copper‑zinc superoxide dismutase; DBP, dibutyl phthalate; DDE, DDE transposase domain; Div, diversin; DSBs, double‑strand breaks; DV, dorsoventral; Dvl, Dishevelled; ERK, extracellular signal‑regulated kinase; FACS, fluorescence‑activated cell sorting; FGF, fibroblast growth factor; FISH, fluorescence in situ hybridization; FoxO, Forkhead box O; fz, frizzled; GABAergic, γ‑aminobutyric acid‑ergic; gH4, germinal histone 4; GO, Gene Ontology; GPx/GPX, glutathione peroxidase; GRN, granulin; GST, glutathione S‑transferase; H&E, hematoxylin and eosin; H3P, phosphorylated histone H3; Hh, Hedgehog; HPA, Human Protein Atlas; ISH, in situ hybridization; KD, knockdown; LCM, laser‑capture microdissection; MATH, meprin and TRAF homology domain; MC‑LR, microcystin‑leucine arginine; MDA, malondialdehyde; MEK, MAPK/ERK kinase; MeRIP‑seq, methylated RNA immunoprecipitation sequencing; ML, mediolateral; MnSOD, manganese superoxide dismutase; mRNA‑seq, messenger RNA sequencing; m⁶A, N⁶‑methyladenosine; myhc, myosin heavy chain; NO, nitric oxide; NOS, nitric oxide synthase; NP, neural progenitor; NPF, neuropeptide F; PAGODA, Pathway and Geneset Overdispersion Analysis; PCP, planar cell polarity; pERK, phosphorylated extracellular signal‑regulated kinase; Pfam, protein family database; PI, propidium iodide; PIWI1, Piwi‑like protein 1; Ptc, patched; qPCR, quantitative polymerase chain reaction; qRT‑PCR, quantitative reverse transcription polymerase chain reaction; RAD51, RAD51 recombinase; Ribo‑seq, ribosome profiling sequencing; RiboMeth‑seq, ribose methylation sequencing; RNA, ribonucleic acid; RNAi, RNA interference; RNA‑seq, RNA sequencing; ROS, reactive oxygen species; rRNA, ribosomal RNA; RT‑qPCR, reverse transcription quantitative polymerase chain reaction; scRNA‑seq, single‑cell RNA sequencing; SEM, scanning electron microscopy; SOD, superoxide dismutase; SYNORF1, synapsin antibody; SYT, synaptotagmin; T‑AOC, total antioxidant capacity; TEM, transmission electron microscopy; TF, transcription factor; TNF, tumor necrosis factor; TRAF, TNF receptor‑associated factor; TUNEL, terminal deoxynucleotidyl transferase dUTP nick end labeling; U0126, a specific MEK inhibitor; UHPLC‑MRM‑MS/MS, ultra‑high‑performance liquid chromatography with multiple reaction monitoring tandem mass spectrometry; VNCs, ventral nerve cords; WISH, whole‑mount in situ hybridization; Wnt, Wingless/Integrated

**Supplementary Figure S1 |** 5-year Moving Average Growth Rate of Publications (From 2000 to 2025)

**Supplementary Figure S1.** The 5-year moving average growth rate of publications (from 2000 to 2025) was analyzed by partitioning the time series into three phases at two nodal points, 2010 and 2020. Prior to 2010, the growth rate exhibited considerable volatility. Between 2010 and 2020, it experienced a sustained decline. Subsequently, from 2020 onward, the growth rate has remained persistently low, even reaching a state of continuous negative growth.

**Reference**

1. Ross KG, Molinaro AM, Romero C, Dockter B, Cable KL, Gonzalez K, et al. SoxB1 Activity Regulates Sensory Neuron Regeneration, Maintenance, and Function in Planarians. Dev Cell. 2018;47(3):331–47.e5. doi: 10.1016/j.devcel.2018.10.014.

2. Currie KW, Pearson BJ. Transcription factors lhx1/5-1 and pitx are required for the maintenance and regeneration of serotonergic neurons in planarians. Development. 2013;140(17):3577–88. doi: 10.1242/dev.098590.

3. März M, Seebeck F, Bartscherer K. A Pitx transcription factor controls the establishment and maintenance of the serotonergic lineage in planarians. Development. 2013;140(22):4499–509. doi: 10.1242/dev.100081.

4. de Sousa N, Rodríguez-Esteban G, Rojo-Laguna JI, Saló E, Adell T. Hippo signaling controls cell cycle and restricts cell plasticity in planarians. PLoS Biol. 2018;16(1):e2002399. doi: 10.1371/journal.pbio.2002399.

5. Molina MD, Saló E, Cebrià F. The BMP pathway is essential for re-specification and maintenance of the dorsoventral axis in regenerating and intact planarians. Dev Biol. 2007;311(1):79–94. doi: 10.1016/j.ydbio.2007.08.019.

6. Scimone ML, Cote LE, Reddien PW. Orthogonal muscle fibres have different instructive roles in planarian regeneration. Nature. 2017;551(7682):623–8. doi: 10.1038/nature24660.

7. Petersen CP, Reddien PW. Wnt signaling and the polarity of the primary body axis. Cell. 2009;139(6):1056–68. doi: 10.1016/j.cell.2009.11.035.

8. Fraguas S, Barberán S, Cebrià F. EGFR signaling regulates cell proliferation, differentiation and morphogenesis during planarian regeneration and homeostasis. Dev Biol. 2011;354(1):87–101. doi: 10.1016/j.ydbio.2011.03.023.

9. Cebrià F, Guo T, Jopek J, Newmark PA. Regeneration and maintenance of the planarian midline is regulated by a slit orthologue. Dev Biol. 2007;307(2):394–406. doi: 10.1016/j.ydbio.2007.05.006.

10. Lapan SW, Reddien PW. Transcriptome analysis of the planarian eye identifies *ovo* as a specific regulator of eye regeneration. Cell Rep. 2012;2(2):294–307. doi: 10.1016/j.celrep.2012.06.018.

11. Altschul SF, Gish W, Miller W, Myers EW, Lipman DJ. Basic local alignment search tool. J Mol Biol. 1990;215(3):403–10. doi: 10.1016/s0022-2836(05)80360-2.

12. Yazawa S, Umesono Y, Hayashi T, Tarui H, Agata K. Planarian Hedgehog/Patched establishes anterior-posterior polarity by regulating Wnt signaling. Proc Natl Acad Sci U S A. 2009;106(52):22329–34. doi: 10.1073/pnas.0907464106.

13. Oderberg IM, Li DJ, Scimone ML, Gaviño MA, Reddien PW. Landmarks in Existing Tissue at Wounds Are Utilized to Generate Pattern in Regenerating Tissue. Curr Biol. 2017;27(5):733–42. doi: 10.1016/j.cub.2017.01.024.

14. Zhen H, Huang M, Zheng M, Gao L, Guo Z, Pang Q, et al. WTAP regulates stem cells via TRAF6 to maintain planarian homeostasis and regeneration. Int J Biol Macromol. 2023;242(Pt 3):124932. doi: 10.1016/j.ijbiomac.2023.124932.

15. Chen J, Li Y, Wang Y, Wang H, Yang J, Pan X, et al. Fibrillarin homologs regulate translation in divergent cell lineages during planarian homeostasis and regeneration. Embo j. 2024;43(24):6591–625. doi: 10.1038/s44318-024-00315-x.

16. Zheng H, Li L, Wang D, Zhang S, Li W, Cheng M, et al. FoxO is required for neoblast differentiation during planarian regeneration. Int J Biol Macromol. 2025;288:138729. doi: 10.1016/j.ijbiomac.2024.138729.

17. Adell T, Cebrià F, Saló E. Gradients in planarian regeneration and homeostasis. Cold Spring Harb Perspect Biol. 2010;2(1):a000505. doi: 10.1101/cshperspect.a000505.

18. Umesono Y, Tasaki J, Nishimura Y, Hrouda M, Kawaguchi E, Yazawa S, et al. The molecular logic for planarian regeneration along the anterior-posterior axis. Nature. 2013;500(7460):73–6. doi: 10.1038/nature12359.

19. Hill EM, Petersen CP. Positional information specifies the site of organ regeneration and not tissue maintenance in planarians. Elife. 2018;7. doi: 10.7554/eLife.33680.

20. Almuedo-Castillo M, Saló E, Adell T. Dishevelled is essential for neural connectivity and planar cell polarity in planarians. Proc Natl Acad Sci U S A. 2011;108(7):2813–8. doi: 10.1073/pnas.1012090108.

21. Swapna LS, Molinaro AM, Lindsay-Mosher N, Pearson BJ, Parkinson J. Comparative transcriptomic analyses and single-cell RNA sequencing of the freshwater planarian Schmidtea mediterranea identify major cell types and pathway conservation. Genome Biol. 2018;19(1):124. doi: 10.1186/s13059-018-1498-x.

22. Suzuki-Horiuchi Y, Schmitz H, Barlassina C, Eccles D, Sinn M, Ortmeier C, et al. Transcription Factors Active in the Anterior Blastema of Schmidtea mediterranea. Biomolecules. 2021;11(12). doi: 10.3390/biom11121782.

23. Cui G, Zhou JY, Ge XY, Sun BF, Song GG, Wang X, et al. m(6) A promotes planarian regeneration. Cell Prolif. 2023;56(5):e13481. doi: 10.1111/cpr.13481.

24. Jin B, Li X, Zhang Q, Zhou W, Liu Y, Dong Z, et al. Toxicity assessment of microcystin-leucine arginine in planarian Dugesia japonica. Integr Zool. 2024;19(6):1135–50. doi: 10.1111/1749-4877.12780.

25. Wu F, Kong Z, Ge P, Sun D, Liu D, Dong Z, et al. Ecotoxicological evaluation and regeneration impairment of planarians by dibutyl phthalate. Environ Res. 2024;257:119403. doi: 10.1016/j.envres.2024.119403.

26. Thiruvalluvan M, Barghouth PG, Tsur A, Broday L, Oviedo NJ. SUMOylation controls stem cell proliferation and regional cell death through Hedgehog signaling in planarians. Cell Mol Life Sci. 2018;75(7):1285–301. doi: 10.1007/s00018-017-2697-4.

27. Forsthoefel DJ, Cejda NI, Khan UW, Newmark PA. Cell-type diversity and regionalized gene expression in the planarian intestine. Elife. 2020;9. doi: 10.7554/eLife.52613.

28. Xie W, Qu J, Xing N, Gao L, Deng H, Liu D, et al. DjCapon Affects the Neural Regeneration and Light Sensitivity via NO and Wnt5a Signaling in Planarian Dugesia japonica. Mol Neurobiol. 2025;62(8):10561–76. doi: 10.1007/s12035-025-04915-4.

29. Imperadore P, Fiorito G. Cephalopod Tissue Regeneration: Consolidating Over a Century of Knowledge. Front Physiol. 2018;9:593. doi: 10.3389/fphys.2018.00593.
